# Upregulation of the interferon-inducible antiviral gene RSAD2 in neuroendocrine prostate cancer *via* PVT1 exon 9 dependent and independent pathways

**DOI:** 10.1016/j.jbc.2025.108370

**Published:** 2025-02-28

**Authors:** Rachel E. Bonacci, Meghan McGill, Nu Thuy Anh Le, Murtaza Barkarar, Colin Finnegan, Maya Wilson, Oluwabusola Ajagbe, Chinedum C. Udekwu, Kathryn Gorski, Jyothi Manohar, Andrea Sboner, Olorunseun O. Ogunwobi

**Affiliations:** 1 Department of Biochemistry and Molecular Biology, Michigan State University, East Lansing, Michigan, USA; 2 Englander Institute for Precision Medicine, Weill Cornell Medicine, New York, New York, USA

**Keywords:** cell biology, prostate cancer, interferon, cellular immune response, long-noncoding RNA, androgen receptor, PVT1, neuroendocrine

## Abstract

PVT1 exon 9 overexpression is a newly uncovered aberration in prostate cancer (PCa). We have previously demonstrated the exon 9 region of PVT1 is overexpressed in some patient PCa tissues and caused development of neuroendocrine prostate cancer (NEPC) *in vitro* and *in vivo*. In this study, we focused on elucidating downstream mechanisms induced by PVT1 exon 9 overexpression with the goal of further understanding its role in NEPC development. RNA-seq analysis of a PVT1 exon 9 overexpressing PCa model revealed significant enrichment of genes responsible for inducing inflammatory processes including RSAD2. We observed RSAD2 overexpression in all NEPC models examined whereas PVT1 exon 9 was overexpressed only in a subset of the NEPC models. We identified two distinct pathways in which RSAD2 is overexpressed: one dependent and one independent on PVT1 exon 9 overexpression. Knockdown of RSAD2 suppressed cell proliferation and migration suggestive of its role as a therapeutic target in NEPC. We identified RSAD2 induces increased cell proliferation, colony formation, and may be involved in the transition between CRPC and NEPC. Distinct differences between PVT1 exon 9–dependent and PVT1 exon 9–independent NEPC models include differences in type II interferon signaling and AR modulation. PVT1 exon 9 binds to RSAD2 protein and disruption of binding significantly impedes downstream interferon gamma secretion by PVT1 exon 9–dependent NEPC cells. These novel findings indicate the importance of these two independent pathways in NEPC, the need to identify relevant NEPC patient populations and study strategies for targeting PVT1 exon 9 and/or RSAD2.

Neuroendocrine prostate cancer (NEPC) is a deadly disease with no effective treatments, high rates of metastasis, low levels of prostate-specific antigen, and poor overall and median survival rates, compared to other subtypes of prostate cancer (PCa). Although NEPC is considered a rare disease compared to castration-resistant prostate cancer (CRPC) or hormone-sensitive prostate cancers, this disease urgently demands research attention as its incidence is predicted to increase 5% annually (1). Furthermore, CRPC and NEPC are intertwined diseases, and it is a well-established clinical phenomenon that a proportion of patients with CRPC treated long-term with androgen deprivation therapeutics, like abiraterone and enzalutamide, develop NEPC (2). This disease is commonly referred to as treatment-induced NEPC (2). Based on the overall poor survival outcomes for NEPC, around 7-months, and a lack of effective treatment strategies, there is a clear and urgent need to identify actionable therapeutic targets for this disease.

Plasmacytoma variant translocation 1 (PVT1) is a long noncoding gene located on the 8q24 chromosomal locus and is involved in a variety of cancer-related processes including activating growth signaling pathways, stimulating angiogenesis, and promoting stem-cell signaling pathways (3, 4, 5). Made up of at least nine distinct exons and encoding six microRNAs, PVT1 is highly complex (6). Full-length PVT1 has been well implicated in the carcinogenesis and progression of PCa (5, 6). We have previously found that exon 9 region of the PVT1 gene is an oncogenic factor in PCa and can act independently from the full-length PVT1 gene (7). Overexpression of PVT1 exon 9 can induce NEPC development from nontumorigenic prostate epithelial cells (7). The downstream mechanisms of action and functions of PVT1 exon 9 remain unclear and the underlying molecular aberrations contributing to NEPC development require further study. Radical S-adenosyl methionine domain containing protein 2 (RSAD2) is a viral response enzyme that has been well studied in virology (human papilloma virus, human immunodeficiency virus, COVID-19) (8, 9, 10, 11, 12, 13, 14, 15). In noncancerous cells, during viral infection, RSAD2 is upregulated and induced mainly by interferon signaling pathways which enhance the activity of T-cells and alter metabolic processes that attack the virus (9, 10). As part of the radical S-adenosylmethionine superfamily, RSAD2 acts as a universal methyl donor and converts CTP to ddCTP, a novel antiviral molecule which inhibits viral transcription (10). RSAD2 can also be induced by infections independent of the interferon pathways including human cytomegalovirus and chikungunya virus (11, 12). Many studies have explored RSAD2 localization as it has been found in a variety of cellular compartments, all of which are dependent on the viral infection present (13, 14, 15). Our current data indicates that RSAD2 is upregulated in PVT1 exon 9 overexpressing NEPC cells and RSAD2 overexpression has clinical relevance. To our knowledge, there have been no previous studies investigating RSAD2 in the context of any PCa subtype.

In this current study, we investigated the role of PVT1 exon 9 and RSAD2 in NEPC with the overarching goal of uncovering whether these molecular aberrations can be exploited as therapeutic targets in NEPC.

## Results

### PVT1 exon 9 overexpression leads to RSAD2 overexpression and is clinically relevant

**Figure 1:**
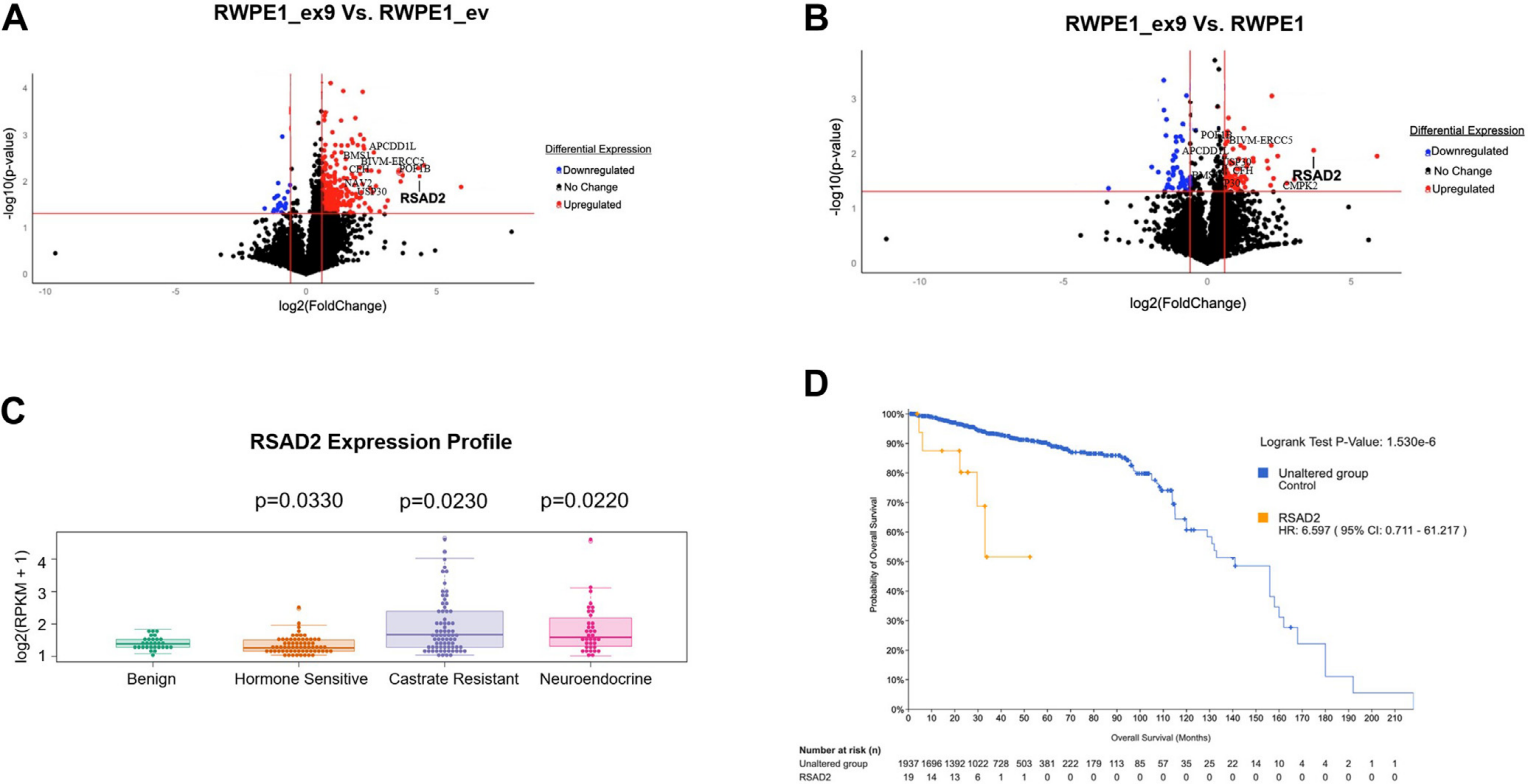
**RNA-seq analysis of PVT1-exon 9 overexpression.***A*, volcano plot of RNA-seq data comparing RWPE1_ex9 *versus* RWPE1_ev. Volcano plot was made in RStudio comparing log_10_(*p*-value) and log2 Fold change. *B*, volcano plot of RNA-seq data comparing RWPE1_ex9 *versus* RWPE1_WT. Volcano plot was made in RStudio comparing log_10_*p*-value and log2 Fold change. *C*, assessment of RSAD2 expression in Weill Cornell Medicine dataset comparing benign (n = 28), hormone-sensitive prostate cancer (n = 66), castrate-resistant prostate cancer (n = 72), and neuroendocrine disease (n = 35). Statistics were provided by collaboration (RShiny application) comparing benign tissue all tumor subtypes. *D*, cBioPortal analysis of all relevant prostate cancer cases comparing survival between RSAD2 altered and nonaltered disease. Statistics were provided by cBioPortal software where significance is indicated with *p*-value <0.05.

Unlike RWPE1 (WT nontumorigenic prostate epithelial cells) and RWPE1_ev (RWPE1 cells stably expressing empty vector) cells, RWPE1_ex9 (RWPE1 cells stably overexpressing PVT1 exon 9) cells formed tumors *in vivo* that have been histologically characterized as small cell (neuroendocrine) PCa subtype (7) suggestive of the role PVT1 exon 9 overexpression plays in NEPC development. To uncover the genes altered by PVT1 exon 9 overexpression and to understand how PVT1 exon 9 overexpression contributes to NEPC development, we performed RNA-seq on RWPE1_ex9 *versus* RWPE1_ev cells and RWPE1_ex9 *versus* RWPE1 cells. RNA-seq data analysis revealed many genes (>100) were differentially expressed exclusively in each comparison while only nine were consistently and significantly differentially expressed in both; all of which were upregulated (*p*-value <0.05, log2FC > 0.5) (Fig 1, A–B, Table 1). Of all the top differentially expressed genes common to both datasets (Table 1), we found RSAD2 to be the most significantly overexpressed based on *p*-value and log_2_ fold change. We wanted to understand whether RSAD2 expression had clinical relevance in any PCa subtype. Analysis of multiple published datasets in cBioPortal revealed RSAD2 was altered in 2% of the PCa cases although there was no differentiation between advanced PCa and neuroendocrine-specific subtype indicating the need for further analysis. To address this, we analyzed gene expression data from whole exome sequencing of prostate tissues of patients seen at Weill Cornell Medicine. We found RSAD2 was significantly overexpressed in both CRPC and NEPC disease compared to benign tissue and hormone-sensitive PCa (*p*-value <0.001) (Fig. 1*C*). We observed that RSAD2 overexpression in PCa, as a whole, contributes to statistically significant poorer overall survival (Fig. 1*D*). Taken together, these data indicate that PVT1 exon 9 overexpression leads to RSAD2 upregulation, and RSAD2 overexpression is a clinically relevant molecular aberration in NEPC that requires further investigation.

**Table 1:** Differentially expressed genes from RNA-seq dataset RWPE1_WT, RWPE1_EV, and RWPE1_ex9

Gene name	Log_2_Fold change ex9 versus WT	*p*-value	Log_2_Fold change ex9 versus EV	*p*-value
RSAD2	4.340	0.008	3.700	0.009
BIVM-ERCC5	2.415	0.038	2.198	0.039
CFH	1.910	0.019	2.074	0.014
APCDD1L	1.883	0.009	1.280	0.027
POF1B	2.493	0.006	2.220	0.007
CMPK2	4.518	0.005	2.297	0.029
NAV2	1.065	0.023	1.256	0.034

Statistics were obtained from students *t* test with 95% confidence interval. Expression was determined significant when *p*-value < 0.05 and log_2_fold threshold was set either greater than 1.00 or less than −1.00.

### PVT1 exon 9 overexpression is induced by the activation of an alternative promoter

**Figure 2:**
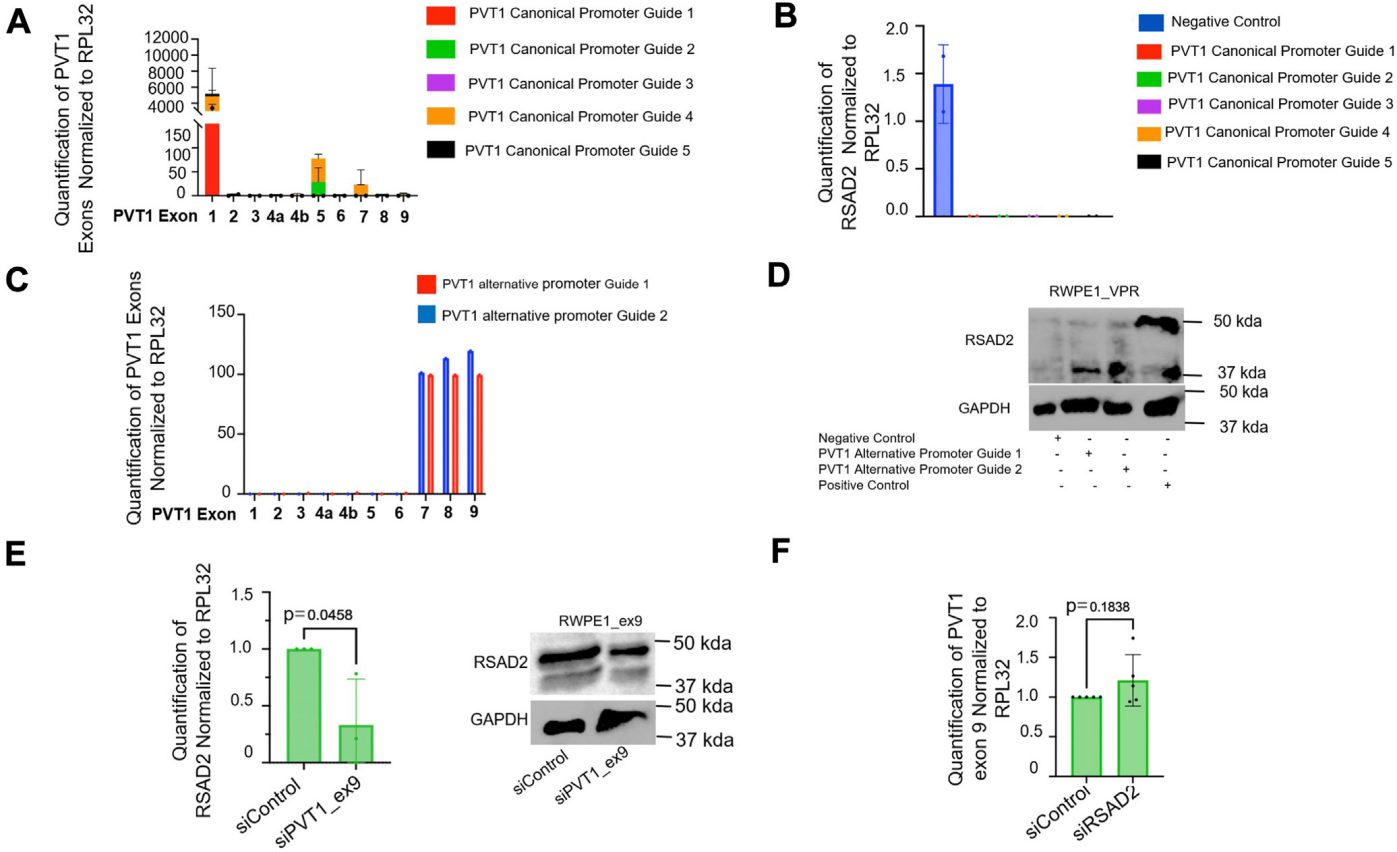
**PVT1 exon 9 overexpression may be caused by alternative promoter site on PVT1 gene.***A*, RT-qPCR expression analysis of individual PVT1 exons from RWPE1_VPR cells transiently treated with five guides that span the PVT1 canonical promoter region (sequences described in Table S1) (two biological replicates). RPL32 and negative control guides were used as normalization controls. *B*, RT-qPCR expression analysis of RSAD2 from RWPE1_VPR cells transiently treated with five guides that span the PVT1 canonical promoter region (sequences described in Table S1) (two biological replicates). RPL32 and negative control guides were used as normalization controls. *C*, RT-qPCR expression analysis of RWPE1_VPR cells transiently treated with two guides that span the PVT1 alternative promoter region assessing each individual exon (two biological replicates). RPL32 and negative control guides were used as normalization controls. *D*, representative Western blot of RWPE1_VPR cells transiently transfected with two guides which span the PVT1 alternative promoter region assessing RSAD2 expression. Positive control cells (RWPE1_ex9) were also included. GAPDH was used as housekeeping gene. *E*, RT-qPCR expression analysis of RWPE1_ex9 cells silenced for PVT1 exon 9 (siRNA) and assessing RSAD2 expression. RPL32 was used as normalization control (three biological replicates). Statistics were provided by PRISM software using two-tailed unpaired *t* test at 95% confidence interval. Representative Western blot of RWPE1_ex9 cells silenced for PVT1 exon 9 (siRNA) and assessing RSAD2 expression. We found loss of RSAD2 expression when PVT1 exon 9 was knocked down. GAPDH was used as housekeeping gene. *F*, RT-qPCR expression analysis of RWPE1_ex9 cells silenced for RSAD2 (siRNA) and assessing PVT1 exon 9 expression (five biological replicates). RPL32 was used as normalization control.

We confirmed the validity of the RWPE1_ex9 model by exhibiting significant overexpression of PVT1 exon 9 (Fig. S1*A*). We have previously observed PVT1 exon 9 is significantly overexpressed in a subset of PCa patient tissues (7) but the question of how PVT1 exon 9 was upregulated remained. We first assessed the structure of the PVT1 gene using the ENSEMBL database which showed that there is one canonical promoter and multiple predicted promoter regions (Fig. S1*B*). We hypothesized that PVT1 exon 9 overexpression may be due to activation of an alternative promoter site compared to the canonical promoter, specifically at the alternative promoter site directly upstream of PVT1 exon 9 (Fig. S1*B*). To determine whether this alternate promoter region may be responsible for PVT1 exon 9 upregulation, we utilized CRISPRa in the RWPE1 nontumorigenic prostate epithelial cell model. The RWPE1 base model was chosen because it has characteristically low baseline expression of PVT1 gene (16) which would reduce background signal. We first treated RWPE1 cells, stably transfected with CRISPRa machinery (RWPE1_VPR), with five guides that spanned the canonical promoter region (Table S1). In all the guides tested, compared to negative control guide, we found PVT1 exon 1, 5, and 7 to be induced (Fig. 2*A*). To further confirm PVT1 exon 9 suppression, we selected RSAD2 expression as a readout. We found no expression of RSAD2 when testing with any of the canonical guides (Fig. 2*B*). Due to the short span of the alternative promoter region, we generated only two guides for testing (Table S1). In either guide tested, compared to negative control, we found activation of PVT1 exon 7, 8, and 9 (Fig. 2*C*). RSAD2 expression was also visualized (Figs. S1*C*, S2*D*). To verify the specificity of RSAD2 upregulation to PVT1 exon 9 overexpression, we activated the alternative promoter region and followed with knockdown of PVT1 exon 9. Compared to scrambled control, we observed loss of RSAD2 expression in the PVT1 exon 9 suppressed cells (Fig. S1, *D* and *E*), which further indicates that PVT1 exon 9 overexpression is responsible for RSAD2 upregulation rather than PVT1 exon 7 or PVT1 exon 8. Together, these data indicate that stimulation of the alternative promoter region may be responsible for the overexpression of PVT1 exon 9.

### PVT1 exon 9 is upstream of RSAD2 and differentially expressed in NEPC models

**Figure 3:**
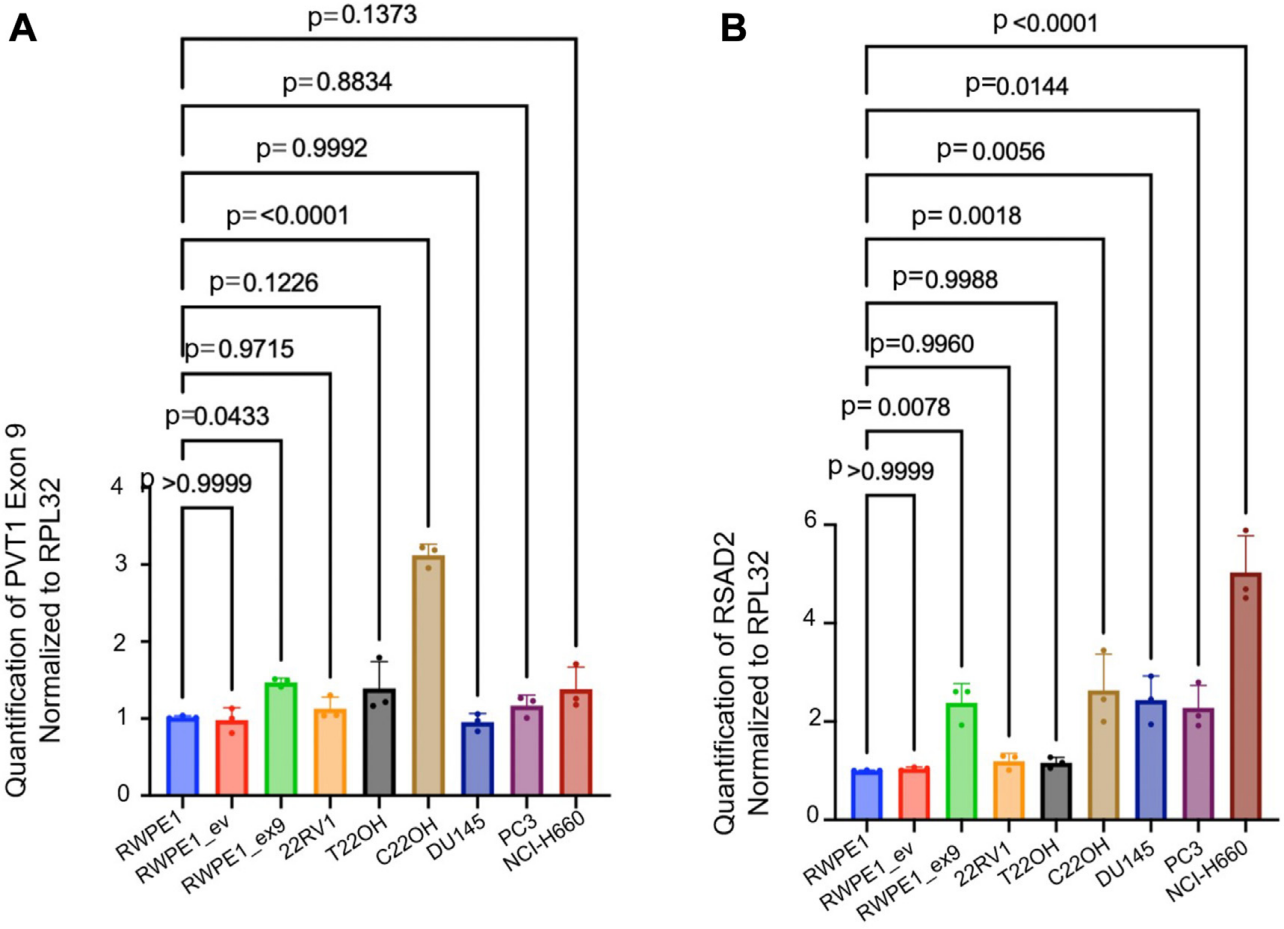
**PVT1 exon 9 and RSAD2 are expressed differentially in NEPC models.***A*, RWPE1, RWPE1_ev, RWPE1_ex9, 22RV1, T22OH, C22OH, DU145, NCI-H660, and PC3 cell lines assessing PVT1 exon 9 expression. Expression was normalized to RPL32 housekeeping gene (three biological replicates). All statistics (*p*-values) were performed using PRISM software using two-tailed unpaired *t* test with 95% confidence interval. *B*, RT-qPCR expression analysis of RWPE1, RWPE1_ev, RWPE1_ex9, 22RV1, T22OH, C22OH, DU145, and PC3 cell lines assessing RSAD2 expression. Expression was normalized to RPL32 housekeeping gene (three biological replicates). All statistical analysis (including *p*-values) were performed using PRISM software using two-tailed unpaired *t* test with 95% confidence interval.

To confirm RSAD2 is downstream of PVT1 exon 9, based on RNA-seq, knockdown of PVT1 exon 9 caused a significant reduction in RSAD2 mRNA and protein expression (Fig. 2*E*), whereas RSAD2 knockdown left PVT1 exon 9 expression unchanged (Fig. 2*F*). Assessment of PVT1 exon 9 expression in a panel of PCa cell lines modeling various clinical subtypes of PCa revealed PVT1 exon 9 overexpression in the PCa cell lines modelling NEPC. We found that the RWPE1_ex9 and C22OH cell models of NEPC had significant upregulation of PVT1 exon 9, compared to RWPE1 (Fig. 3*A*). The C22OH cell line (a circulating tumor cell line) and the T22OH cell line (derived from the dissociation of primary tumor) are both derived from *in vivo* tumor growth that resulted from implantation of the 22RV1 PCa cell line into mice (17). Western blotting analysis of 22RV1, T22OH, and C22OH revealed that C22OH overexpresses classic markers of NEPC, including chromogranin A and synaptophysin (Fig. S2*A*) while the parental CRPC model T22OH had less expression of chromogranin A and no synaptophysin. We further hypothesized high RSAD2 expression would be present in only the RWPE1_ex9 and C22OH models; due to its correlation with PVT1 exon 9 overexpression. Contrary to our hypothesis, we observed all NEPC cell models exhibited higher expression of RSAD2 than RWPE1 (Fig. 3*B*). Based on these data, we have determined there are two differing pathways in which RSAD2 is upregulated in NEPC; one because of PVT1 exon 9 overexpression and the other independent of PVT1 exon 9 overexpression caused by an unknown mechanism.

### RSAD2 may be involved in the transition between CRPC and NEPC

Due to the clear and established evidence linking NEPC development from CRPC (2), we tested the hypothesis that RSAD2 may be a factor in the transition from CRPC to NEPC. To examine whether RSAD2 is involved in the transition between CRPC and NEPC, we analyzed the GSE199596 GEO2.0 dataset. We found that RSAD2 expression was higher (fold change 2.15, *p*-value <0.05) in transitional CRPC (Fig. S2*B*) than classical NEPC disease and was less expressed in CRPC.

### PVT1 exon 9 regulates RSAD2 mRNA stability

To determine the interaction between PVT1 exon 9 and RSAD2, we first assessed whether there is a RNA:RNA interaction between PVT1 exon 9 and RSAD2. Using BLAST sequence alignment, we assessed the entire RSAD2 DNA and mRNA structure (promoter region, coding sequence, 5′UTR, gene body, 3′UTR), for binding with PVT1 exon 9 and found no significant evidence predicting that these entities bind (Fig. S3*A*). There was one region that modeling indicated may be binding at the 3′UTR but a dual luciferase assay we performed indicated no specific binding between these sequences (Fig. S3*B*). To assess whether PVT1 exon 9 interacts with RSAD2 in a post-transcriptional manner, we assessed the mRNA decay rate of RSAD2. We found that in PVT1 exon 9–overexpressed cells (RWPE1_ex9, C22OH), there was a higher stability of RSAD2 mRNA than the RSAD2 overexpressing model (PC3, RWPE1_RSAD2) (Fig. S3*C*) suggestive of an indirect role PVT1 exon 9 plays in RSAD2 stability post-transcriptionally.

### PVT1 exon 9 binds directly to RSAD2 protein

**Figure 4:**
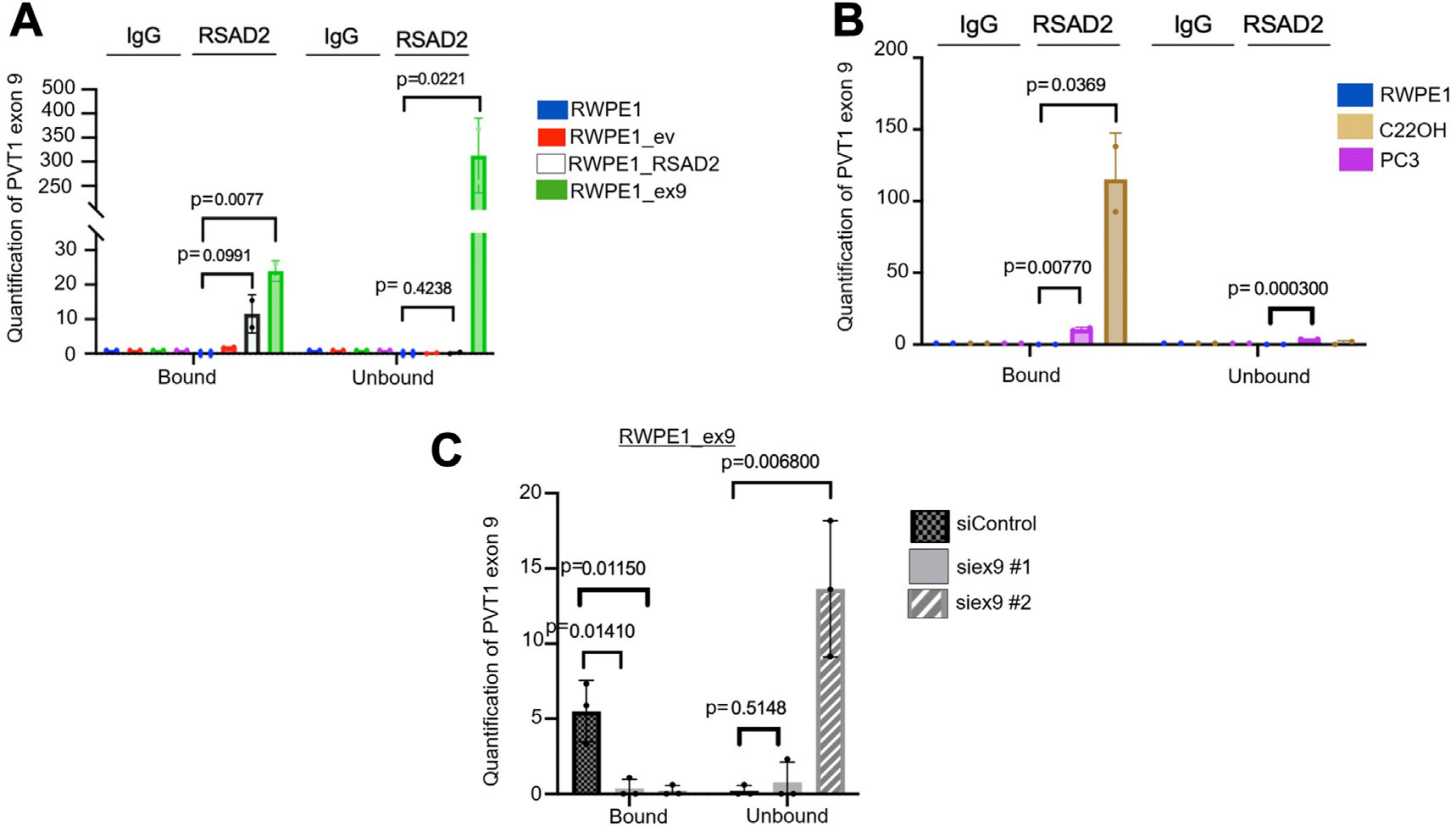
**PVT1 exon 9 binds to RSAD2 protein.***A*, RNA-immunoprecipitation experiment assessing baseline PVT1 exon 9 after RSAD2 pulldown using RT-qPCR in RWPE1, RWPE1_ev, RWPE1_RSAD2, and RWPE1_ex9 cell models (two biological replicates). Fold change of PVT1 exon 9 after RNA-immunoprecipitation, either bound or unbound, was calculated based on initial cell RNA-input expression PVT1 exon 9. PRISM software provided *p*-values using two-sided unpaired *t* test at 95% confidence interval. *B*, RNA-immunoprecipitation experiment assessing baseline PVT1 exon 9 after RSAD2 pulldown using RT-qPCR in RWPE1, C22OH, and PC3 cell models (two biological replicates). Fold change of PVT1 exon 9 after RNA-immunoprecipitation, either bound or unbound, was calculated based on initial cell RNA-input expression PVT1 exon 9. PRISM software was used to perform statistical analysis and determine *p*-values using two-sided unpaired *t* test at 95% confidence interval. *C*, RNA-immunoprecipitation experiment assessing RWPE1_ex9 cells treated with either siControl or siPVT1 exon 9 (siex9 #1, siex9 #2), using two differing siRNAs (three biological replicates). Fold change of PVT1 exon 9 after RNA-immunoprecipitation, either bound or unbound, was calculated based on initial cell RNA-input expression PVT1 exon 9. PRISM software provided *p*-values using two-sided unpaired *t* test at 95% confidence interval.

We also investigated the RNA:protein interaction between PVT1 exon 9 and RSAD2 protein. Computational modeling using RPIseq revealed that PVT1 exon 9 binds to RSAD2 protein (0.75 and 0.75 where < 0.5 indicates positive interaction). To determine experimentally if PVT1 exon 9 binds to RSAD2 protein, we performed RNA-immunoprecipitation in the PVT1 exon 9–dependent and PVT1 exon 9–independent models. We found that PVT1 exon 9 is bound to RSAD2 protein much more so in RWPE1_ex9 (PVT1 exon 9–dependent) than in RWPE1_RSAD2 (PVT1 exon 9–independent) (Figs. 4*A*, Fig. S4*A*). To further confirm this, we utilized other NEPC cell lines that either have PVT1 exon 9 overexpression (C22OH) or do not (PC3) and found the same binding pattern (Figs. 4*B*, Fig. S4*B*). We then knocked down PVT1 exon 9, using two siRNAs that target differing regions of PVT1 exon 9, one near the 5′ end of PVT1 exon 9 and the other near the 3′ end of PVT1 exon 9 in the RWPE1_ex9 cell line, and found that binding to RSAD2 protein was significantly disrupted (Fig. 4*C*). The siRNA siex9 #1 (3′end) showed degradation of PVT1 exon 9, whereas siRNA siex9 #2 (5′ end) showed recovery of PVT1 exon 9 in the unbound fraction (Fig. 4*C*). Based on the secondary structure of PVT1 exon 9 (Fig. S4*C*), there are two regions on the gene that are likely binding candidates whereas the rest of the molecule is fixed in secondary hairpin structures (Fig. S4*C*). Analysis of the sequence alignment revealed that there is a positive interaction (>0.5) with the region covered by siRNA ex9 #2 and a negative interaction (<0.5) with the region covered by siRNA ex9 #1 (Fig. S4*C*).

### RSAD2 overexpression induces proliferation and colony formation in prostate epithelial cells

**Figure 5:**
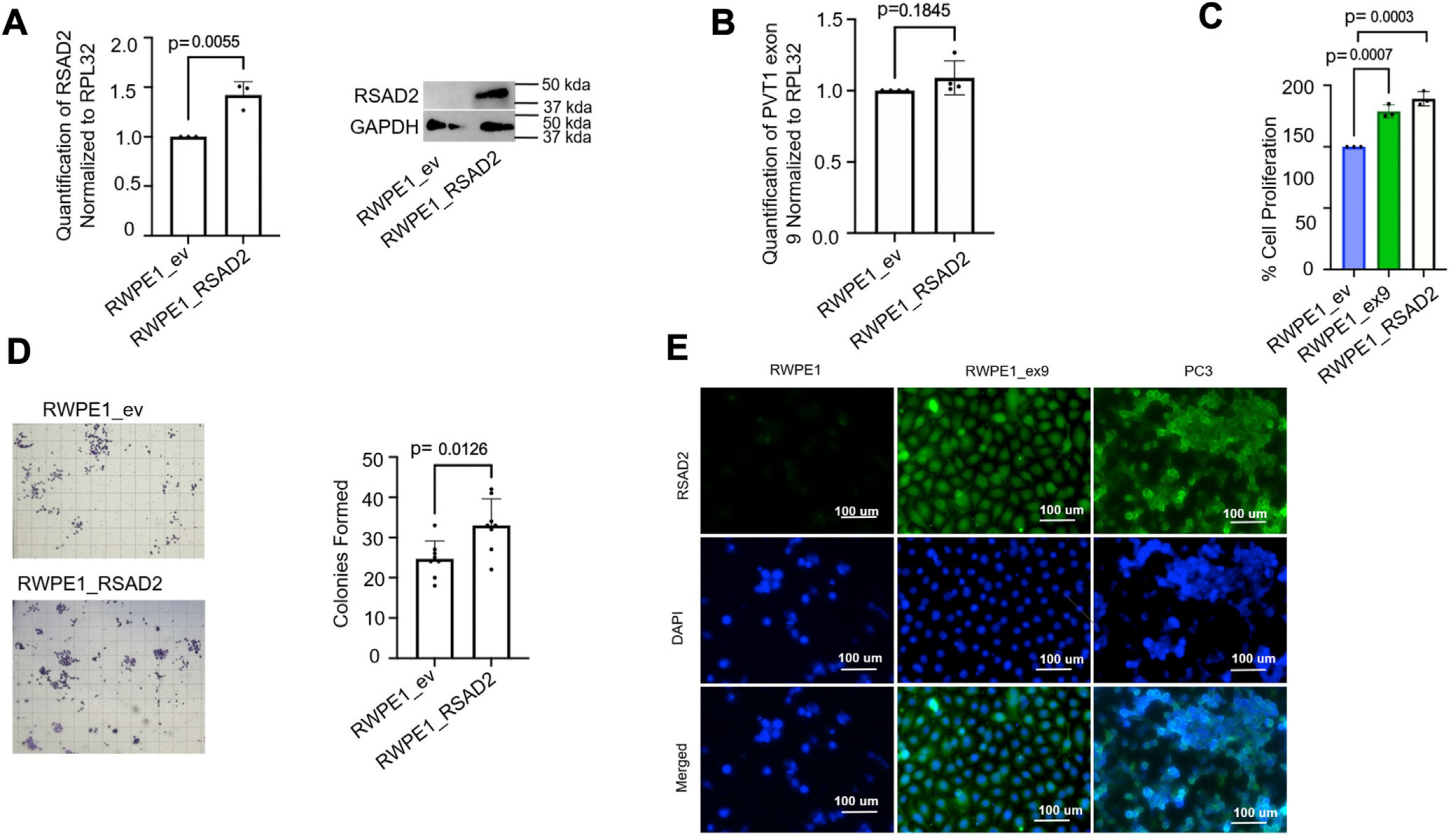
**RSAD2 overexpression promotes cell growth in normal prostate epithelial cells.***A*, validation of RSAD2 overexpression with RNA (three biological replicates) and representative protein in the RWPE1_RSAD2 generated cell line. PRISM software was used to perform statistical analysis and determine *p*-values using two-sided unpaired *t* test at 95% confidence interval. *B*, knockdown of RSAD2 in RWPE1_RSAD2 cell line showed no significant difference in PVT1 exon 9 expression suggesting RSAD2 overexpression does not impact PVT1 exon 9 overexpression and is downstream of this molecular aberration (three biological replicates). PRISM software was used to perform statistical analysis and determine *p*-values using two-sided unpaired *t* test at 95% confidence interval. *C*, cell proliferation assay assessing differences between RWPE1_ev, RWPE1_ex9, and RWPE1_RSAD2. We found similar changes in cell proliferation between RWPE1_ex9, which was previously published (7), and RWPE1_RSAD2 (n = 2). PRISM software was used to perform statistical analysis and determine *p*-values using two-sided unpaired *t* test at 95% confidence interval. *D*, colony formation assay (n = 3) comparing RWPE1_EV to RWPE1_RSAD2 cell line. We found significantly more colonies in the RWPE1_RSAD2 model than RWPE1_EV suggesting its potential for RSAD2 upregulation inducing pathogenic changes. PRISM software was used to perform statistical analysis and determine *p*-values using two-sided unpaired *t* test at 95% confidence interval. *E*, immunofluorescence of RSAD2 was obtained for RWPE1, RWPE1_ex9 (PVT1 exon 9 dependent), and PC3 (PVT1 exon 9 independent) cell lines, 4′,6-diamidino-2-phenylindole staining for nuclei staining and merged image of the two. Images were taken at 40x magnification.

To understand the phenotypic consequences of RSAD2 overexpression, we overexpressed RSAD2 in the RWPE1 nontumorigenic prostate epithelial cell model (Fig. 5*A*). We found that overexpression of RSAD2 alone does not induce PVT1 exon 9 overexpression (Fig. 5*B*), indicating that RSAD2 overexpression is not also upstream but only downstream of PVT1 exon 9 overexpression. We further observed that overexpression of RSAD2 significantly induced increased cell proliferation to an extent comparable to that caused by overexpression of PVT1 exon 9 (Fig. 5*C*) (7). In addition, it also significantly induced increased colony formation (Fig. 5*D*). UniProt database suggests RSAD2 exists in a variety of cellular compartments and multiple isoforms exist based on studies with normal (nonmalignant) cells (13, 14, 15). To investigate cellular localization of RSAD2 in NEPC, we assessed the PVT1 exon 9–associated and PVT1 exon 9–independent cell models. We observed RSAD2 localization mainly within the nucleus of PVT1 exon 9–dependent models and cytoplasmic localization, observable by the characteristic honeycomb feature, in the PVT1 exon 9–independent models (Fig. 5*E*).

### RSAD2 may be a therapeutic target in PVT1 exon 9–dependent and PVT1 exon 9–independent neuroendocrine PCa

**Figure 6:**
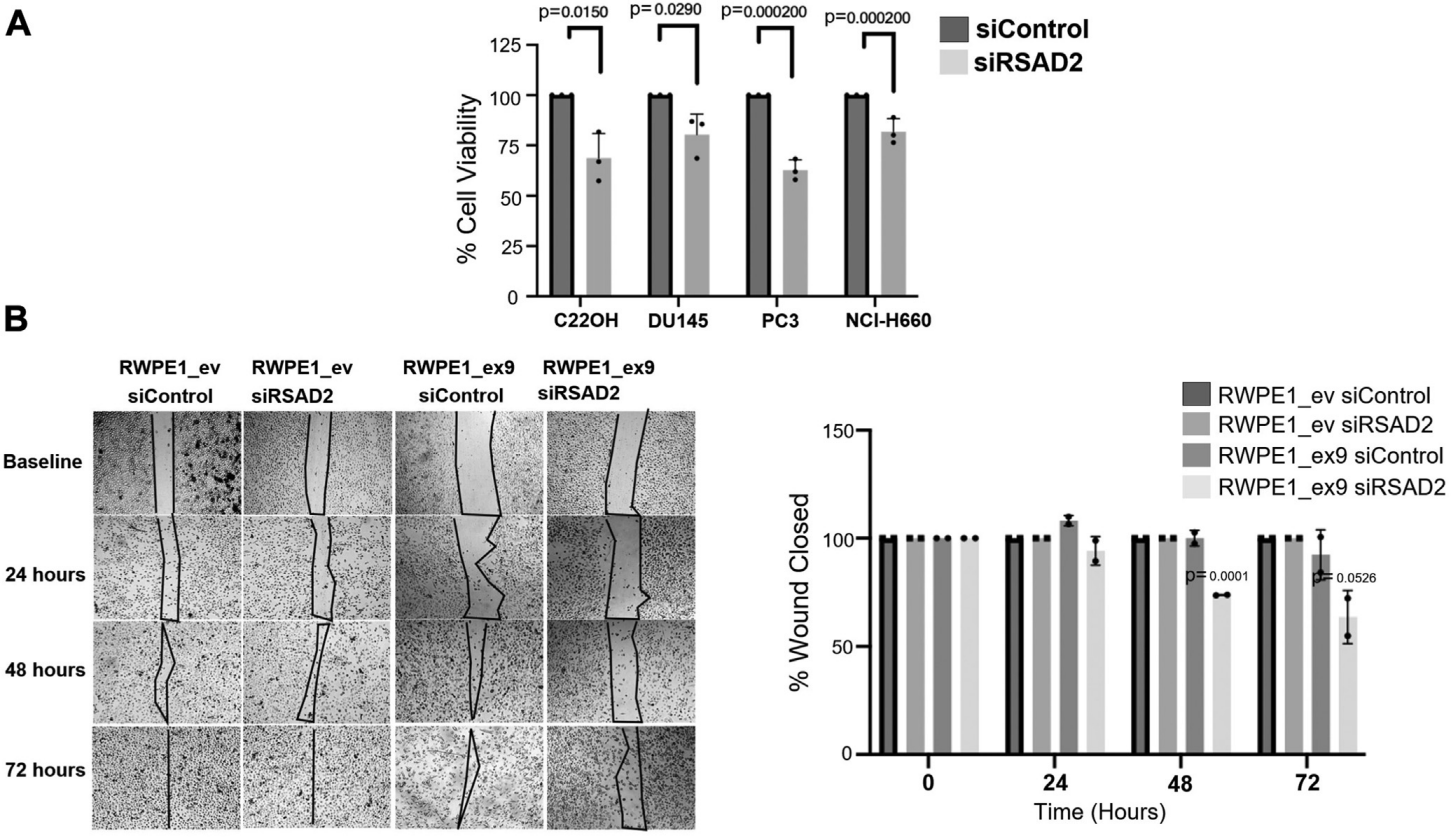
**RSAD2 knockdown leads to loss of cell viability and colony formation.***A*, MTT cell viability assay comparing silenced scrambled control *versus* siRSAD2 treatment in C22OH, DU145, and PC3 cell lines (three biological replicates). PRISM software was used for *p*-value using two-sided *t* test at 95% confidence interval. *B*, wound healing assay assessing whether knockdown of RSAD2 leads to decreased wound healing in RWPE1_ex9 compared to RWPE1_ev cell line (two biological replicates). PRISM software was used to perform statistical analysis and determine *p*-values using two-sided unpaired *t* test at 95% confidence interval.

Knockdown of *RSAD2* in both PVT1 exon 9–dependent and PVT1 exon 9–independent RSAD2 overexpression NEPC models revealed significant inhibition of cell viability and migration (as determined by wound healing assays) suggestive of its role as a potential therapeutic target in this disease (Fig. 6, *A*–*B*).

### PVT1 exon 9 overexpression in NEPC models leads to inflammatory characteristics

**Figure 7:**
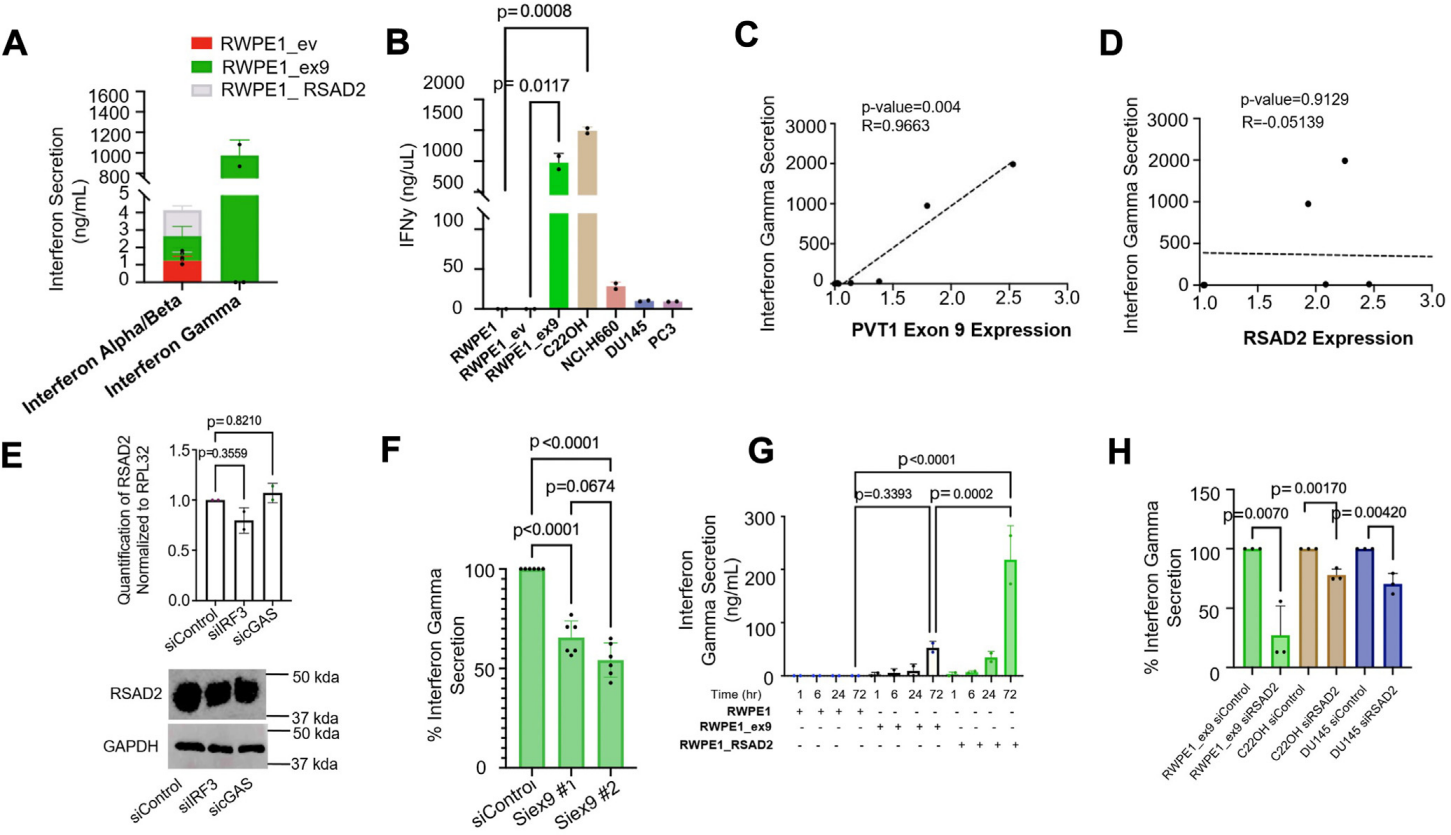
**RSAD2 upregulation stimulates type II interferon signaling mechanisms.***A*, normalization of the type I and II interferon gamma secretion of RWPE1_ex9, RWPE1_RSAD2, and RWPE1_EV cell lines using ELISA assay (two biological replicates). *B*, normalization of the interferon gamma secretion of RWPE1_WT, RWPE1_EV, RWPE1_ex9, C22OH, NCI-H660, DU145, and PC3 cell lines using ELISA assay (two biological replicates). PRISM software provided statistics using two-sided *t* test at 95% confidence interval. *C*, linear regression analysis of interferon gamma secretion and RT-qPCR expression of PVT1 exon 9 from all individual cell lines tested (representative of three biological replicates). PRISM software was used for linear regression analysis, using best fit-line, and provided the statistics and correlation coefficient. *D*, linear regression analysis of interferon gamma secretion and RT-qPCR expression of RSAD2 from all individual cell lines tested. PRISM software was used for linear regression analysis, using best fit-line, and provided *p*-value and correlation coefficient. *E*, knockdown of IRF3 and cGAS in RWPE1_RSAD2 cell line (two biological replicates) does not cause any significant change in RSAD2 mRNA expression. Representative Western blot of RWPE1_RSAD2 cell line with scrambled control, IRF3, or cGAS knockdown and showed no difference in RSAD2 protein expression in any condition. PRISM software was used for *p*-value using two-sided *t* test at 95% confidence interval. *F*, normalized secretion of interferon gamma using ELISA assay of silenced scrambled control *versus* siPVT1 exon 9 knockdown in RWPE1_ex9 cells using either siRNA #1 or siRNA #2 (two biological replicates). PRISM software provided *p*-values using two-way ANOVA with alpha 0.05. *G*, interferon gamma secretion was significantly increased at 72 h in the RWPE1_ex9 model and was increased somewhat in the RWPE1_RSAD2 to a lesser degree (two biological replicates). These results are consistent with our initial findings (Fig. 7*A*) which show substantially increased interferon gamma secretion in the RWPE1_ex9 model. PRISM software provided *p*-values using two-way ANOVA with alpha 0.05. *H*, normalized secretion of interferon gamma using ELISA assay of silenced scrambled control *versus* siRSAD2 knockdown RWPE1_ex9, C22OH, and DU145 cells (three biological replicates). PRISM software provided *p*-values using two-sided student's *t* test at 95% confidence interval.

RSAD2 has been observed as both a type I and II interferon response gene (9, 10) and is able to control secretion of interferon molecules. Full-length PVT1 was observed to be an inhibitor of type I interferon signaling in some cancer models (18, 19). Based on these prior data, we wanted to investigate whether PVT1 exon 9 expression differentially regulates either type I or type II interferon pathways. We observed that in the PVT1 exon 9 and RSAD2 overexpression models, there were low levels of interferon alpha and beta (Fig. 7*A*) but very high levels of secreted interferon gamma only in the PVT1 exon 9 overexpression model (Fig. 7*A*). This finding was consistent with immunofluorescence staining which revealed a higher intensity of intracellular interferon gamma localized within the cytosolic space (Fig. S5*A*) of the PVT1 exon 9 overexpression model. These findings correlate with the other NEPC models that either overexpress PVT1 exon 9 or do not (Figs. 7*B*, Fig. S5*B*). A significantly positive correlation was identified between PVT1 exon 9 expression and interferon gamma secretion (Fig. 7*C*) and no observable correlation was observed between RSAD2 expression and interferon gamma secretion (Fig. 7*D*). These findings suggest interferon gamma secretion is unique to PVT1 exon 9 overexpression in NEPC models, and this aberration likely contributes to protumor promoting processes (20). Because the literature suggests full-length PVT1 suppresses type I interferon signaling in tumor models (18, 19), we wanted to explore whether PVT1 exon 9 also elicits this phenotype. We assessed baseline protein expression of the inflammatory response genes interferon regulatory factor 3 (IRF3) and cyclic GMP-AMP synthase (cGAS) in the PVT1 exon 9 and RSAD2 overexpression models. We found no observable change in either IRF3 or cGAS expression when RSAD2 is overexpressed, whereas PVT1 exon 9 overexpression resulted in the loss of both of these proteins (Fig. S5, *C* and *D*), which correlates with the published literature. Knockdown of either *IRF3* or *cGAS* in the RSAD2 overexpression model produced no dramatic changes in RSAD2 expression at the mRNA level or protein level (Fig. 7*E*). These results suggest RSAD2 expression is not mediated by type I interferon signaling or cGAS/IRF3 signaling. To test whether PVT1 exon 9:RSAD2 binding leads to interferon gamma signaling, we treated the PVT1 exon 9–dependent models (RWPE1_ex9 and C22OH) with siRNA #1 and siRNA #2 and assessed interferon gamma secretion after 48 h (Fig. 7*F*). We found significant loss of interferon gamma secretion with both siRNAs which further indicates that the PVT1 exon 9:RSAD2 interaction is important in PVT1 exon 9–dependent downstream action.

### RSAD2 is regulated differentially based on PVT1 exon 9 expression

Because there are differing levels of secreted interferon gamma in the PVT1 exon 9–independent and PVT1 exon 9–dependent models (Fig. 7*A*), we wanted to understand the rate of secretion and how that differed between PVT1 exon 9 overexpressed and non-overexpressed models. We observed substantial secretion of interferon gamma at 72 h (Fig. 7*G*) in PVT1 exon 9–overexpressing cells; although the secreted amount differed, the rate of secretion did not differ between PVT1 exon 9– and RSAD2-overexpressing cells. Throughout the 72 h time period, we observed no changes in interferon alpha and beta secretion in our PVT1 exon 9 and RSAD2 overexpression models supporting the notion that type I interferon signaling is not relevant to RSAD2 expression (Fig. S5*E*). RSAD2 expression has been previously shown to be an interferon response gene (8, 9, 10, 11, 12, 13, 14, 15) and it also can modulate the secretion of interferon signaling molecules (21)^.^ We observed paracrine signaling from PVT1 exon 9–overexpressing cells was able to induce RSAD2 expression in a non-RSAD2–expressing cell but RSAD2 overexpression alone is not sufficient to induce RSAD2 expression in a non-RSAD2–expressing cell (Fig. S5*F*). We further observed that knockdown of RSAD2 contributed to a reduced secretion of interferon gamma (Fig. 7*H*) which suggests that interferon gamma is downstream of the PVT1 exon 9–RSAD2 or RSAD2 signaling axis.

### PVT1 exon 9 overexpression regulates androgen receptor expression

**Figure 8:**
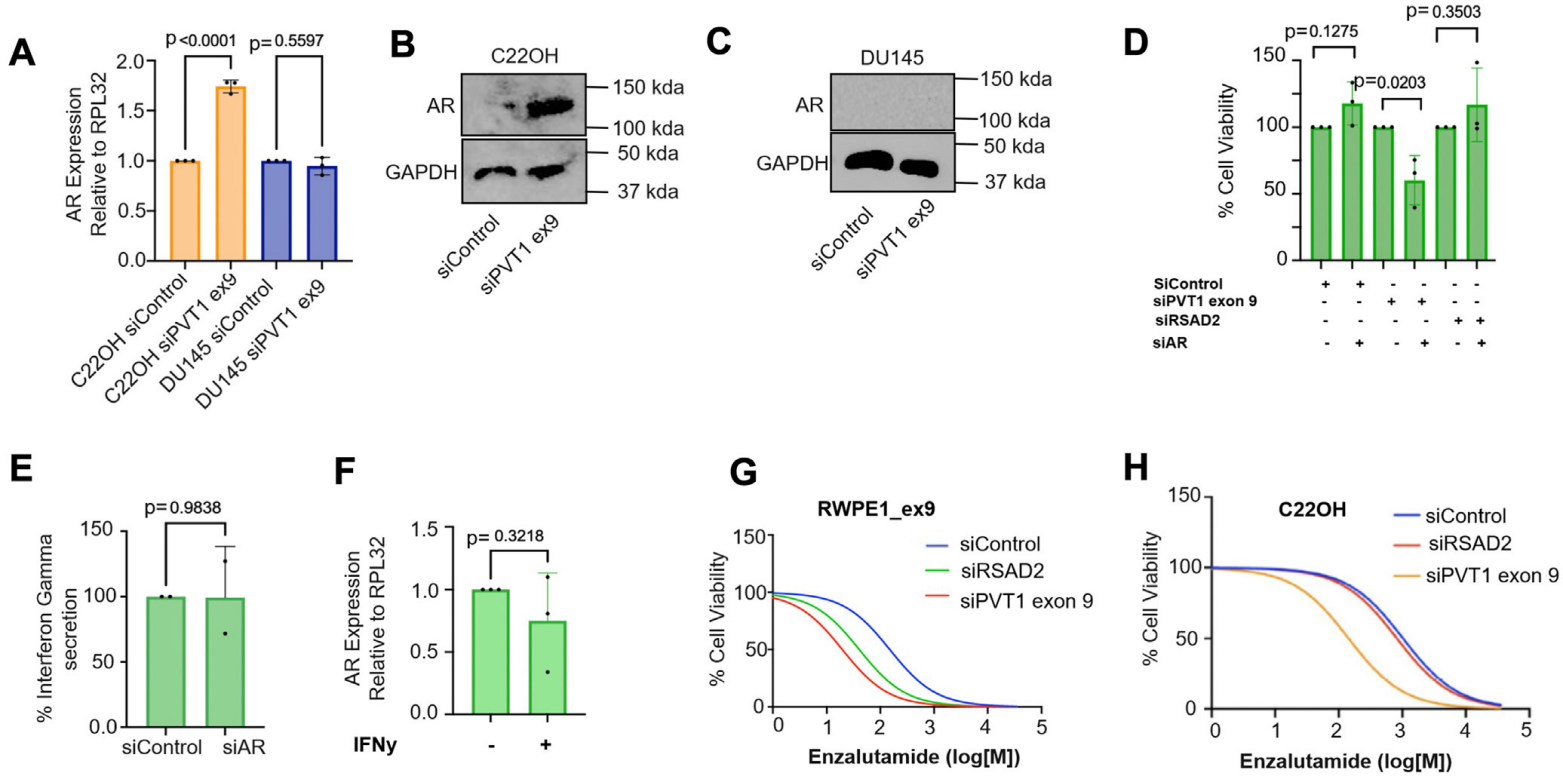
**PVT1 exon 9 contributes to AR suppression in NEPC models.***A*, RT-qPCR analysis of silenced scrambled control *versus* siPVT1 exon 9 in C22OH and DU145 cell lines (three biological replicates). Expression was normalized to RPL32 housekeeping gene. PRISM software was used for *p*-value using two-sided *t* test at 95% confidence interval. *B*–*C*, representative Western blotting of C22OH and DU145 assessing AR expression between silenced scrambled control and siRSAD2 as well as housekeeping genes GAPDH. *D*, cell viability assay assessing knockdown of either scrambled control, PVT1 exon 9, or RSAD2 alone or in combination with AR (three biological replicates). *E*, ELISA analysis of lysates collected from siControl or siAR (48 h timepoint) assessing interferon gamma secretion (two biological replicates) was found to not be significantly altered in either condition. PRISM software was used for *p*-value using two-sided *t* test at 95% confidence interval. *F*, RT-qPCR analysis of RWPE1_ex9 cell line treated with 100 μg/ml of exogenous interferon gamma and assessed for AR expression using RT-qPCR (three biological replicates). Expression was normalized to RPL32 housekeeping gene. PRISM software was used for *p*-value using two-sided *t* test at 95% confidence interval. *G*-*H*, RWPE1_ex9 and C22OH cell lines were treated with increasing doses of enzalutamide (10 nM–10 μM) for 72 h with either siControl, siRSAD2, or siPVT1 exon 9. Cells were processed for proliferation compared to normal control using MTT reagent (three biological replicates). All statistics were provided by PRISM software.

It has been established that NEPC has low expression of androgen receptor (AR) protein (1, 2), and modulation of AR activity is a critical aberration relevant to PCa development, pathogenesis, and treatment. Few studies have investigated the connection between interferon signaling and androgen receptor activity in prostate models (22). In noncancerous prostate epithelial cells, a positive relationship between androgen receptor expression and type-I interferon signaling molecules occurs, but not with type-II interferon signaling molecules (23, 24). Since we observed universally unchanged levels of type I interferon molecules in all models tested (Figs. 7*A*, Fig. S5*B*) including the normal prostate epithelial cell model RWPE1, we hypothesized it was unlikely that type I interferon signaling molecules contributed to modulation of AR in NEPC. Due to the positive correlation found between interferon gamma and PVT1 exon 9, as well as literature evidence suggesting PVT1 interacts with AR to suppress its activity (22), we hypothesized overexpression of PVT1 exon 9 is inversely correlated with AR activity in the PVT1 exon 9–overexpressed NEPC models and AR suppression may be caused by interferon gamma. To address this, we began by performing knockdown experiments to understand whether PVT1 exon 9 modulates AR suppression. We found that in the C22OH model, knockdown of PVT1 exon 9 induced both mRNA and protein expression of AR, whereas the DU145 model, which does not express high levels of PVT1 exon 9, did not have this same phenotype (Fig. 8, *A*–*C*) suggesting an inverse relationship between PVT1 exon 9 and AR expression. Since RSAD2 is downstream of PVT1 exon 9, we wanted to explore whether knockdown of RSAD2 could elicit the same effect. We found that knockdown of RSAD2 mimics what we saw with PVT1 exon 9 knockdown in the C22OH and DU145 models at the mRNA level only (Fig. S6*A*). We utilized our RWPE1_ex9 model for further validation. Upon knockdown in the RWPE1_ex9 cell line, we found that knockdown of PVT1 exon 9 only in combination with knockdown of AR caused statistically significant reduced cell viability (Fig. 8*D*) compared to knockdown of scrambled control or RSAD2. ELISA analysis on the lysates, assessing interferon gamma, showed no significant observable change in interferon gamma secretion between scrambled control or silenced AR (Fig. 8*E*) Finally, treatment with exogenous interferon gamma did not cause increase in AR expression (Fig. 8*F*).

### Knockdown of PVT1 exon 9 enhances sensitivity to enzalutamide

We wanted to understand whether these observations could translate to sensitizing NEPC cell models to clinically relevant AR inhibitors (25, 26, 27). We first observed that the cell lines differed in response to enzalutamide while C22OH, which has the highest quantification of PVT1 exon 9, is the most resistant to enzalutamide (Fig. S6*B*). In the RWPE1_ex9 model, only knockdown of PVT1 exon 9 caused significantly enhanced sensitivity to enzalutamide (Fig. 8*G*) and we found this to also be the case in the C22OH PVT1 exon 9–overexpressed model (Fig. 8*H*). Additionally, in NEPC models that only overexpress RSAD2 (Fig. S6, *C* and *D*), we found knockdown of PVT1 exon 9 led to no notable change in sensitivity to enzalutamide.

## Discussion

**Figure 9:**
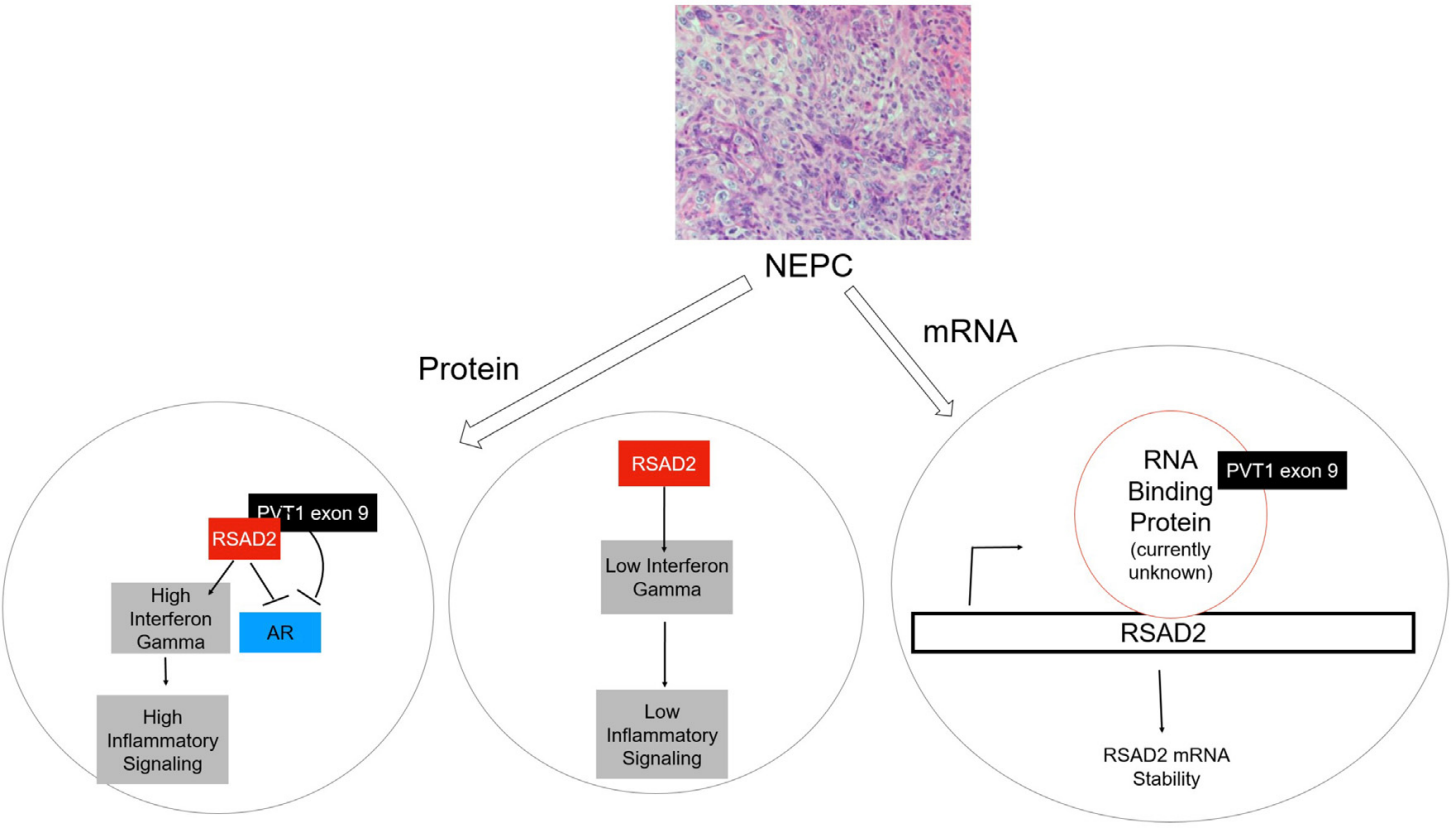
**Schematic of neuroendocrine prostate cancer subsets which overexpress RSAD2 alone or in conjunction with PVT1 exon 9.** The image of neuroendocrine prostate cancer (NEPC) was reused from Pal *et al.*, 2019 *(Genes)* (7).

Here, we report that RSAD2 overexpression is present in NEPC models and is caused by two differing pathways (Fig. 9). These results are significant as there are few notable molecular aberrations known that have potential targetability in NEPC. These results also highlight an alternative way in which AR suppression may differentially occur in NEPC and brings forth potential novel therapeutic targets which require further exploration. The data uncovered from this study underscore the importance of understanding the NEPC patient populations that demonstrate both PVT1 exon 9 and RSAD2 overexpression or those that only overexpress RSAD2 in their PCas. It is important to identify and stratify these subsets of patients in order to understand and explore novel treatment strategies, such as whether immune checkpoint inhibitors may have utility in NEPC patient populations with tumors that demonstrate PVT1 exon 9/RSAD2-dependent interferon gamma signaling. Our findings are clinically relevant as we observed interferon gamma signaling is driven by high PVT1 exon 9 expression, and PVT1 exon 9–overexpressed NEPC models have more inflammatory characteristics compared to only RSAD2-overexpressed models. It is also important to understand whether these pathways are present in any other neuroendocrine malignancies. Recent research has found that RSAD2 upregulation is present in colorectal cancer and is a prognostic biomarker for immunotherapy (28). This suggests that RSAD2 is not restricted to neuroendocrine disease but may be present in a variety of malignancies and its potential as a therapeutic target may be far-reaching.

There are no currently known available therapeutics that can actively target the RSAD2 protein. It is possible that direct targeting of the RSAD2 protein, or mRNA, alone or in combination with other clinically available therapeutic agents could lead to better treatment outcomes in NEPC. This approach could lead to insights into not only ways to target RSAD2 in malignant diseases but in other diseases where RSAD2 is predicted to be a therapeutic target (28). We observed RSAD2 overexpression in NEPC may be in differing cellular compartments and have differing functions based on underlying PVT1 exon 9 expression. This needs to be explored further to understand how to best utilize therapeutic strategies to target RSAD2 or PVT1 exon 9.

Furthermore, work is needed to understand the functionality of the RSAD2 protein as we have noted there are a variety of isoforms present which may depend on PVT1 exon 9 overexpression status. We further aim to understand the indirect interaction between PVT1 exon 9 and RSAD2 through possible intermediary binding partners such as polyadenylate-binding protein cytoplasmic 3 (PABPC3), which has been predicted to bind to both RSAD2 and PVT1 exon 9 mRNAs. We found evidence that PVT1 exon 9 overexpression leads to suppression of androgen receptor expression in PVT1 exon 9/RSAD2-overexpressed NEPC cells. We noted that although RSAD2 is a regulator of androgen receptor expression, we found that PVT1 exon 9 likely plays a much larger role in androgen receptor re-expression as observed. The interaction between RSAD2 and AR that we observed may be due to residual or compensatory activity of being downstream of PVT1 exon 9. It is clear PVT1 exon 9 overexpression may play a greater role in AR inhibitor resistance beyond loss of innate AR function in NEPC, and non-overexpressed PVT1 exon 9 cells have a differing mechanism related to AR suppression.

Further, AR suppression in PVT1 exon 9–overexpressed cells does not affect downstream interferon gamma secretion. It is necessary to pursue further study to test whether targeting both PVT1 exon 9 and RSAD2 in NEPC would open the door to beneficially targeting AR rather than PVT1 exon 9 alone. Targeting RSAD2 alone may hold therapeutic benefit in a proportion of NEPC patients as well as patients that have transitional CRPC to NEPC disease. Although we did not find that knockdown of RSAD2 had the ability to resensitize NEPC cells to androgen receptor inhibitors, it is possible that identifying novel therapeutic strategies to inhibit RSAD2 is still critical to achieving better outcomes in NEPC.

A major unanswered clinical question is the well-established higher incidence and prevalence of aggressive PCa and worse outcomes from PCa in the men of African ancestry (29, 30). We have previously demonstrated that PVT1 exon 9 overexpression may be linked to racial disparities in NEPC (16). These current data may provide the much-needed rationale to study the PVT1 exon 9/RSAD2 overexpression pathway in PCa in the men of African ancestry. It is important to identify whether the PVT1 exon 9/RSAD2 overexpression pathway is relevant in PCa in all racial groups or whether it is particularly more relevant in PCa only in specific population groups such as men of African ancestry. These studies may lead to further explanations as to why men of African ancestry experience a higher incidence and prevalence of aggressive PCa and worse outcomes from PCa than men of other racial groups.

## Experimental procedures

### Cell culture

The human PCa cells RWPE1, NCI-H660, 22RV1, DU145, and PC3 cells were obtained from American Type Culture Collection. The RWPE1_ev and RWPE1_ex9 cells were derivatives of RWPE1, methods for overexpression can be found in previous publication (7). C22OH and T22OH cell lines are derivatives of the 22RV1 cell line that can be found in previous publication (17). RWPE1, RWPE1_ev, RWPE1_ex9, RWPE1_RSAD2 and RWPE1_VPR cells were cultured in keratinocyte serum-free media supplemented with human epidermal growth factor and bovine serum as previously published (7). DU145 cells were cultured in EMEM media. PC3 cells were cultured in F-12K media. NCI-H660 cells were cultured in HITES media and 22RV1, C22OH, and T22OH cell lines was cultured in RPMI-1640 media. All cell lines were cultured with 5 to 10% fetal bovine serum with 1% penicillin/streptomycin (Gibco, Thermo Fisher Scientific) at 37 °C with 5% CO2. All commercially available cell lines were purchased from American Type Culture Collection and characterized for the absence of potential contaminants.

### siRNA transfection and reagents

Cells were transfected with either scrambled control (siControl), siRSAD2 or siPVT1 exon 9 #1 or siPVT1 exon 9 #2, using Lipofectamine 3000 (Thermo Fisher Scientific) according to the manufacturer's instructions. Cells were plated in 6-well plates 6 to 24 h prior to transfection in regular media. The siRNA constructs were added at a final concentration of 0.75 μg in OPTI-MEM media. Regular media was removed, and cells were incubated with siRNA–lipofectamine complexes for 6-h in OPTI-MEM then regular media was added to the cells and incubated for 24 to 72 h depending on the individual experiment. Knockdown validation was performed using RNA (real-time quantitative PCR, RT-qPCR) and protein (Western blot) verification.

### Establishment of PVT1 exon 9 and RSAD2 overexpression models

RWPE1_ex9 and RWPE1_ev cells were generated from previously published methods (7). RWPE1_RSAD2 cells were generated from ORF RSAD2 lentiviral particles purchased from Horizon Discovery (OHS5900-202621272) and transfected according to standard protocol. Cells were visualized from GFP tag and selected using puromycin-supplemented media (150 ng/ml) for one-month for stable selection. RWPE1_VPR cells were generated from lentiviral particles that transfect the dCas9-VPR CRISPRa machinery with hEF1a promoter (Horizon Discovery, VCAS11922) according to standard protocol and selected using puromycin-supplemented media (150 ng/ml) for one month based on preliminary RWPE1 kill curve experiments for stable selection.

### RNA sequencing library preparation and sequencing analysis

Total RNA was extracted from RWPE1, RWPE1_ev and RWPE1_ex9 cells using RNeasy isolation kit (Qiagen) per the manufacturer's instructions. RNA was sent to Arraystar who performed the RNA sequencing experiment. For library preparation, the NEBNext Poly(A) mRNA Magnetic Isolation Module (New England Biolabs) RiboZero Magnetic Gold Kit (Human/Mouse/Rat) (Epicenter, an Illumina Company) KAPA Stranded RNA-Seq Library Prep Kit (Illumina) was used followed by NanoDrop ND-1000 quantification. Agarose electrophoresis was used to check the integrality of total RNA samples. One to two micrograms of total RNA of each sample was taken for RNA-seq library preparation. Briefly, mRNA was isolated from total RNA with NEBNext Poly (A) mRNA Magnetic Isolation Module. rRNA was removed from the total RNA with a RiboZero Magnetic Gold Kit. The enriched mRNA or rRNA-depleted RNA was used for RNA-seq library preparation using KAPA Stranded RNA-Seq Library Prep Kit (Illumina). The completed libraries were qualified on Agilent 2100 Bioanalyzer for concentration, fragment size distribution, between 400 and 600 bp, and adapter dimer contamination. The amount was determined by absolute quantification qPCR method. The barcoded libraries were mixed in equal amounts and used for sequencing on the instrument. The DNA fragments in well mixed libraries were denatured with 0.1 M NaOH to generate single-stranded DNA molecules, loaded onto channels of the flow cell at 8 pM concentration, and amplified *in situ* using NovaSeq 6000 S4 Reagent Kit (300 cycles). Sequencing was carried out using the Illumina NovaSeq 6000 according to the manufacturer’s instructions. Sequencing was carried out by running 150 cycles. Raw sequencing data generated from Illumina NovaSeq 6000 that pass the Illumina chastity filter are used for following analysis. Trimmed reads (trimmed 5′, 3′-adaptor bases) are aligned to reference genome. Based on alignment statistical analysis (mapping ratio, rRNA/mtRNA content, fragment sequence bias), we determine whether the results can be used for subsequent data analysis. If so, the expression profiling, differentially expressed genes, and differentially expressed transcripts are calculated. The novel genes and transcripts are also predicted. Principal component analysis, correlation analysis, hierarchical clustering, Gene Ontology, pathway analysis, scatter plots, and volcano plots are performed for the differentially expressed genes in R or Python environment for statistical computing and graphics.

### Sequence Alignment using BLAST

mRNA sequences of RSAD2 were separated based on functional subunit (promoter region, coding sequence, 5′UTR, exon, 3′UTR, promoter region) by utilizing University of California Santa Cruz (UCSC) genome browser (CRCh38/hg38). The individual sequences were compared to PVT1 exon 9 sequence using BLAST.

### PCa tissue RNA-seq analysis

Whole transcriptome sequencing (RNA-seq) and subsequent data processing was carried out in accordance with the procedures outlined in the referenced studies (31, 32). Bulk RNA was isolated from frozen samples for RNA-seq using the Promega Maxwell 16 MDx device (Maxwell 16 LEV simplyRNA Tissue Kit, catalog number AS1280). The specimens were prepared for RNA-seq using either the TruSeq RNA Library Preparation Kit v2 or riboZero, as previously described (33). The integrity of the RNA was confirmed using the Agilent Bioanalyzer 2100 from Agilent Technologies. The total RNA was then converted into cDNA using Superscript III from Invitrogen. The sequencing was carried out on GAII, HiSeq 2000, or HiSeq 2500 platforms as paired-ends (31, 32, 33). All reads were independently aligned using STAR_2.4.0f1 (34) for sequence alignment against the human genome sequence build hg19, which was downloaded *via* the UCSC genome browser. SAMTOOLS v0.1.19 (35) was used for sorting and indexing the reads. The expression values (FPKMS) were estimated using Cufflinks (2.0.2) (36), and the GENCODE v19 (37) GTF file was used for annotation.

### Real-time quantitative PCR

RNA was extracted from RWPE1, LNCAP, 22RV1, T22OH, C22OH, DU145, PC3, NCI-H660, RWPE1_ev, and RWPE1_ex9 cells using RNeasy isolation kit (Qiagen, 74104) per manufacturer's instructions. The extracted RNA was reverse transcribed using High-Capacity cDNA reverse transcription kit (Thermo Fisher Scientific, 4368814) following the manufacturer's instructions. RT-qPCR was then conducted on a Quant-studio 3 Real Time PCR machine (Thermo Fisher Scientific) using PowerUp SYBR RT-qPCR Master Mix (Thermo Fisher Scientific, A25741). RPL32 was used as the internal reference gene. The primer sequences designed for the qPCR assay are in Table S2. Experiments were performed with, at minimum, two technical replicates per biological replicate. The data were analyzed using the 2-▵ ▵Ct method with RPL32 serving as a standard gene for normalization.

### RNA decay

The indicated cells (RWPE1, RWPE1_ex9, C22OH, PC3, RWPE1_ev) were plated in 6-well plate at a confluency of 80% prior to treatment. Cells were treated with 1 μM of flavopiridol in fresh media and collected at 1, 2, and 5 h as well as untreated (0 h) timepoint. RNA was extracted, and RSAD2 expression was assessed using RT-qPCR. PRISM software was used to model half-life and provide curve fitting.

### RNA secondary structure analysis

The secondary structure of PVT1 exon 9 was determined using Vienna RNAfold WebServer: http://rna.tbi.univie.ac.at/cgi-bin/RNAWebSuite/RNAfold.cgi.

### RNA–protein interaction prediction tool

We utilized the Iowa State University RNA-Protein Interaction Prediction tool: http://pridb.gdcb.iastate.edu/RPISeq/ to determine predictive binding of protein sequence and RNA sequence. Positive predictive binding is indicated by a value >0.5.

### RNA immunoprecipitation

RWPE1, RWPE1_ev, RWPE1_ex9, RWPE1_RSAD2, C22OH, and PC3 cells were seeded and collected at ∼75% confluency and processed according to established protocols and Abcam published protocol (38, 39). Lysates were treated with 3 μl IgG control antibody or RSAD2 polyclonal antibody overnight at 4 °C with gentle agitation and separated based on bound fraction (with beads) or unbound fraction. Initial fraction (5%) was collected and processed for RNA and Western blotting. RNA was converted to cDNA and then analyzed using RT-qPCR for PVT1 exon 9 while protein was quantified and ran using Western blotting. Silenced RWPE1_ex9 cells were also treated in the same process after 48 h incubation with siRNAs.

### Cell proliferation studies

All indicated cells were plated in 96-well clear flat bottom plates 6 to 24 h prior to treatment or transfection. Once adhered, or settled, cells were treated with siRNA constructs or compounds and incubated at 37 °C with 5% CO2 for 72 h. After 72-h incubation, 5 μl of 10 mg/ml stock solution of 3-(4,5-Dimethylthiazol-2-yl)-2,5-diphenyltetrazolium bromide (Sigma Aldrich, 475989) or 5 μL of 10 mg/ml stock solution of WST-1 (Sigma Aldrich, 5015944001) was added to the media of each well and incubated at 37 °C with 5% CO2 for 2 h. After visualization of 3-(4,5-Dimethylthiazol-2-yl)-2,5-diphenyltetrazolium bromide uptake, media was aspirated and cells were lysed with 100% isopropanol alcohol and mixed for 10 min to visualize the formazan granules. MTT or WST-1 plates were read on Varioskan Lux spectrophotometer (Thermo Fisher Scientific) at 470 nm. Two technical replicates, at minimum, were performed for each biological replicate.

### Colony formation

RWPE1_EV and RWPE1_RSAD2 cells were plated in 6-well plates at a density of 1000 cells per well in regular media. Once adherent, regular media was replaced every 3 to 4 days until sufficient colonies could be visualized in either group. Cells were fixed with 100% methanol and stained with crystal violet for 5-min. Plates were washed with deionized water until extra dye was removed. Colonies were visualized under standard microscope. In order to quantify, we drew three representative squares, three technical replicates, 1 cm each, per well, and counted (40). Colony counts were plotted using PRISM software and students two-sided *t* test was used for statistics with 95% confidence interval.

### Enzyme-linked immunosorbent assay

The supernatants of baseline or knockdown NEPC cells, plated at ∼80% confluence, were collected at various timepoints as indicated in the individual experiments. Supernatants were spun down and clarified. Interferon gamma and alpha/beta kit (Thermo Fisher Scientific) was utilized to quantify secretion. Experiments were performed with two technical replicates, at minimum, per biological replicate.

### Supernatant transfer experiments

RWPE1 cells were plated at a confluence of ∼60% in regular media in 12-well plates. Supernatant from RWPE1_ex9 or RWPE1_RSAD2 cells were collected 24 and 72 h postincubation. Media was spun down and mixed in 1:1 ratio with regular SFM media and added to RWPE1 cells or regular SFM media was added to the cells. Cells were incubated for 72 h and harvested for protein.

### Western blotting

Cells were first plated in a 6-well plate 12 to 24 h before treatment at 75% confluence density in regular supplemented media. Media was removed and cells were treated with the indicated siRNAs for 24 h, then supplemental regular media was added for an additional 48-h. After incubation, cells were collected, pelleted by centrifugation, and lysed with M-PER Mammalian Protein Extraction Reagent (Thermo Fisher Scientific, 78501). Cell lysates were frozen at −80 °C 1-h before quantification. To quantify the cellular lysates, the extracts were thawed, spun down, and supernatant collected. Protein was quantified using Bradford assay with 2 mg/ml bovine serum albumin (BSA) (Thermo Fisher Scientific, 23209) and 1:1 diluted standards in protein extraction reagent. Equal amounts of protein were loaded into cast 8% Bis-Acrylamide gel with 2x Laemmli reducing SDS blue sample buffer (Thermo Fisher Scientific, J60015.AC) supplemented with 4% beta-mercaptoethanol to break remaining disulfide bonds. Samples were boiled for 7-min at 75 °C before loading. The gel was ran according to standard protocols. The gel was transferred, using standard methods, onto either a 0.45 um nitrocellulose membrane or PDVF membrane for 1 h in a 4 °C cold room at constant 100 voltage in transfer buffer (Tris/Glycine buffer (BioRad) 10x diluted with deionized water and 20% methanol final concentration). After transfer, the membrane was blocked with 5% nonfat dried milk dissolved in PBS + 0.1% Tween-20 (TBST) for 20 to 30 min. Primary rabbit antibody (anti-RSAD2, anti-AR, anti-Synaptophysin, anti-GAPDH) or primary mouse antibody (anti-chromogranin A) was added at a concentration of 1:1000 in TBST for 1 h at room temperature or overnight in cold room with constant agitation. After primary antibody incubation, the membrane was washed three times for 10 min with TBST on orbital shaker. Anti-rabbit secondary antibody (Cell Signaling, 7074S) containing horseradish peroxidase tag was added at a concentration of 1:2000 in TBST for 45-min at room temperature. After secondary antibody incubation, the membrane was washed three times for 10-min each with TBST on orbital shaker. The membranes were visualized using a 1:1 solution of chemiluminescence substrate solution (Cytiva Life Sciences) on Western blotting imaging machine (Azure Biosystems).

### Immunofluorescence

RWPE1, RWPE1_ev, RWPE1_RSAD2, DU145, PC3, RWPE1_ex9 cells were plated on Nunc Lab-Tek II chambered slide system at ∼75% confluence for 12 to 24 h before experiments in regular media. After adherence was visualized, media was removed and cells were fixed with 100% cold acetone for 5-min. The slides were blocked with 5% BSA (Sigma, A3294) for 20 min at room temperature. The rabbit anti-RSAD2 or anti-interferon gamma antibody (Thermo Fisher Scientific, PA5-106337; Cell Signaling, 8455) was applied at a concentration of 1:500 in 5% BSA for 1 h at room temperature. Subsequently, Alexa Fluor 488 or Alexa 647 anti-rabbit secondary antibody (Invitrogen, A32731; Invitrogen, A-31573) was used at a concentration of 1:1000 in 5% BSA. The nuclei were counterstained with 4′,6-diamidino-2-phenylindole (Thermo Fisher Scientific) and coverslip was placed over the slides. The images were acquired using an EVOS M7000 microscope (Thermo Fisher Scientific) and taken at 40x magnifications.

### Analysis of publicly available databases

GEO dataset GSE199596 was accessed from GEO repository and assessed with GEO2R. Analysis consisted of stratifying samples provided by the dataset and underlying publication (41). Samples consisted of PDX tumor models of varying histologies, either castrate resistant or neuroendocrine. We classified these samples based on the underlying stated characteristics: (1) classic neuroendocrine disease (AR negative, NE positive), (2) transitional neuroendocrine disease (AR positive, NE positive), (3) classic castrate-resistant disease (AR positive, NE negative). With GEO2R analysis, we obtained the log_2_ fold change and *p*-values between the groups indicated within the manuscript for RSAD2 expression. ENSEMBL database was used to assess promoter regions of the PVT1 gene (ENSG00000249859). cBioPortal was used to access previously published TCGA, Memorial-Sloane Kettering, Broad-Cornell, Multi-institute, Fred Hutchinson (CRC, SU2C/PCF, MCTP, CPC-GENE, SMMU) using PCa cohort selection tool on PCa or aggressive PCa cases with only copy number changes of RSAD2. The cBioPortal generated survival data and calculated hazard ratios. Underlying racial data was assessed in each individual study (when annotated).

### Gene Ontology analysis

We analyzed the inclusive significant upregulated genes from RNA-seq analysis RWPE1_EV *versus* RWPE1_ex9 and RWPE1 *versus* RWPE1_ex9 using The Gene Ontology Resource Database, url: https://geneontology.org/. We assessed the biological processes using the homo sapiens dataset.

### Statistical analysis

The statistical analyses employed in this study included the student's *t* test, two-way ANOVA, and Pearson correlation coefficient performed using PRISM software as well as using The Cancer Genome Atlas and GEO2.0 database. The individual statistical tests used are indicated in figure legends. Data are presented as the mean ± SD and exact *p*-values are indicated.

## Data availability

Data are to be shared upon request, please contact corresponding author for inquiries.

## Supporting information

This article contains supporting information.

## Conflicts of interest

O. O. O. is the co-founder of NucleoBio Inc, a start-up biotechnology company. All other authors declare that they have no conflicts of interest with the contents of this article.
