# Supplementary material for: Upregulation of the interferon-inducible antiviral gene RSAD2 in neuroendocrine prostate cancer via PVT1 exon 9 dependent and independent pathways
Source: J Biol Chem. 2025 Feb 28;301(4):108370. doi: 10.1016/j.jbc.2025.108370 (PMC11994405; doi:10.1016/j.jbc.2025.108370)
Supplement: Figure S1 [file mmc1.pdf]

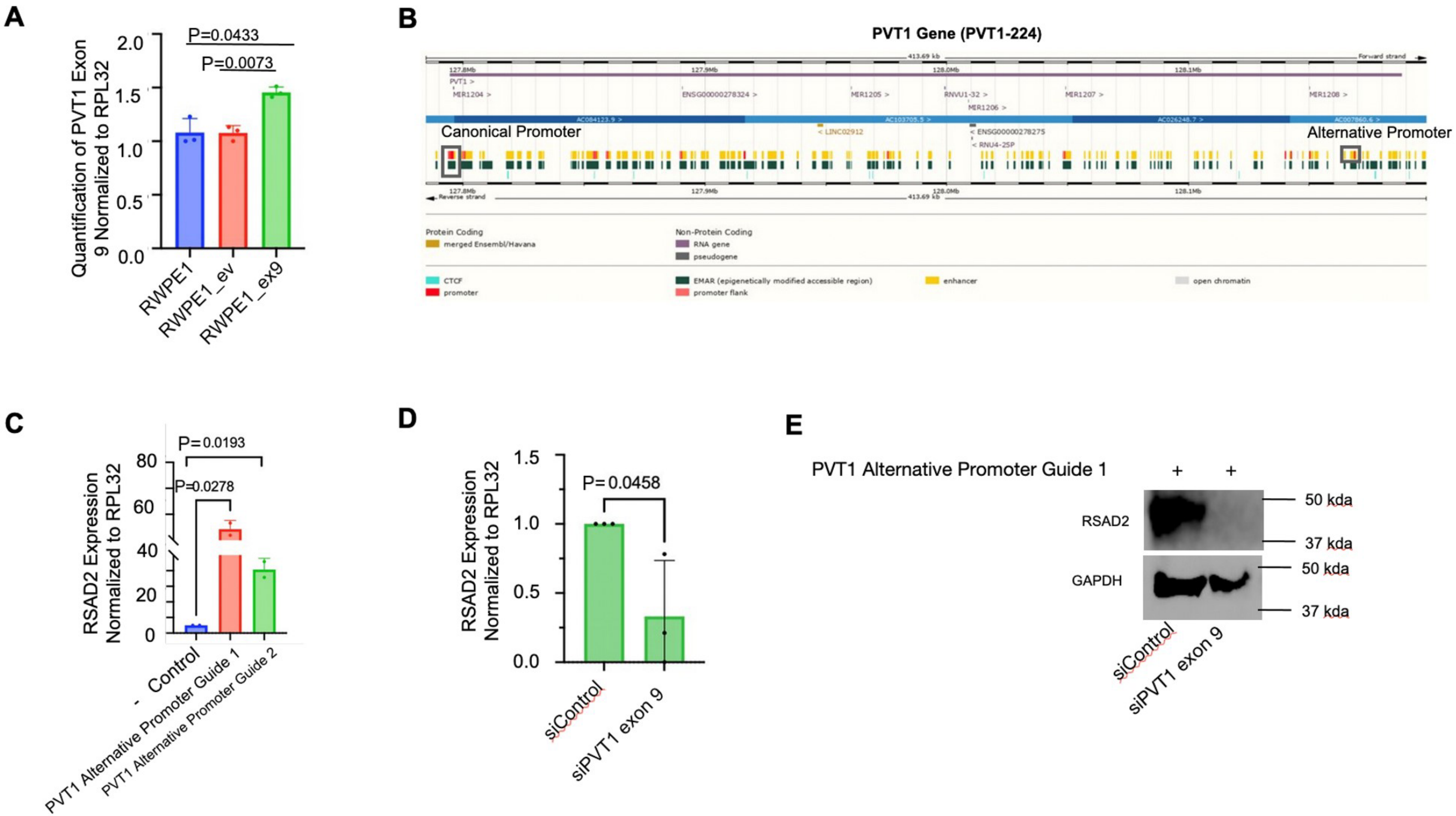


**Supplemental Figure 1. PVT1 exon 9 upregulation validation. [A]** RT-qPCR results which verify that PVT1 exon 9 is overexpressed in RWPE1_ex9 model compared to RWPE1_WT and RWPE1_ev (three biological replicates). Statistics provided by PRISM software using paired t-test at 95% confidence interval. **[B]** ENSEMBL assessment of PVT1 gene and annotated promoter regions. **[C]** RWPE1_VPR treated with guide 1 and 2 (stimulation of PVT1 alternative promoter region) revealed upregulation of RSAD2 mRNA (two biological replicates). Statistics provided by PRISM software using paired t-test at 95% confidence interval. **[D-E]** Knockdown of RWPE1_VPR treated with guide 1 (stimulation of PVT1 alternative promoter region) followed by knockdown with either scrambled control or siPVT1 exon 9 revealed loss of RSAD2 mRNA (three biological replicates) and representative protein image. Statistics were provided by PRISM software, unpaired t-test at 95% confidence interval.
