# Supplementary material for: Upregulation of the interferon-inducible antiviral gene RSAD2 in neuroendocrine prostate cancer via PVT1 exon 9 dependent and independent pathways
Source: J Biol Chem. 2025 Feb 28;301(4):108370. doi: 10.1016/j.jbc.2025.108370 (PMC11994405; doi:10.1016/j.jbc.2025.108370)
Supplement: Figure S2 [file mmc2.pdf]

A

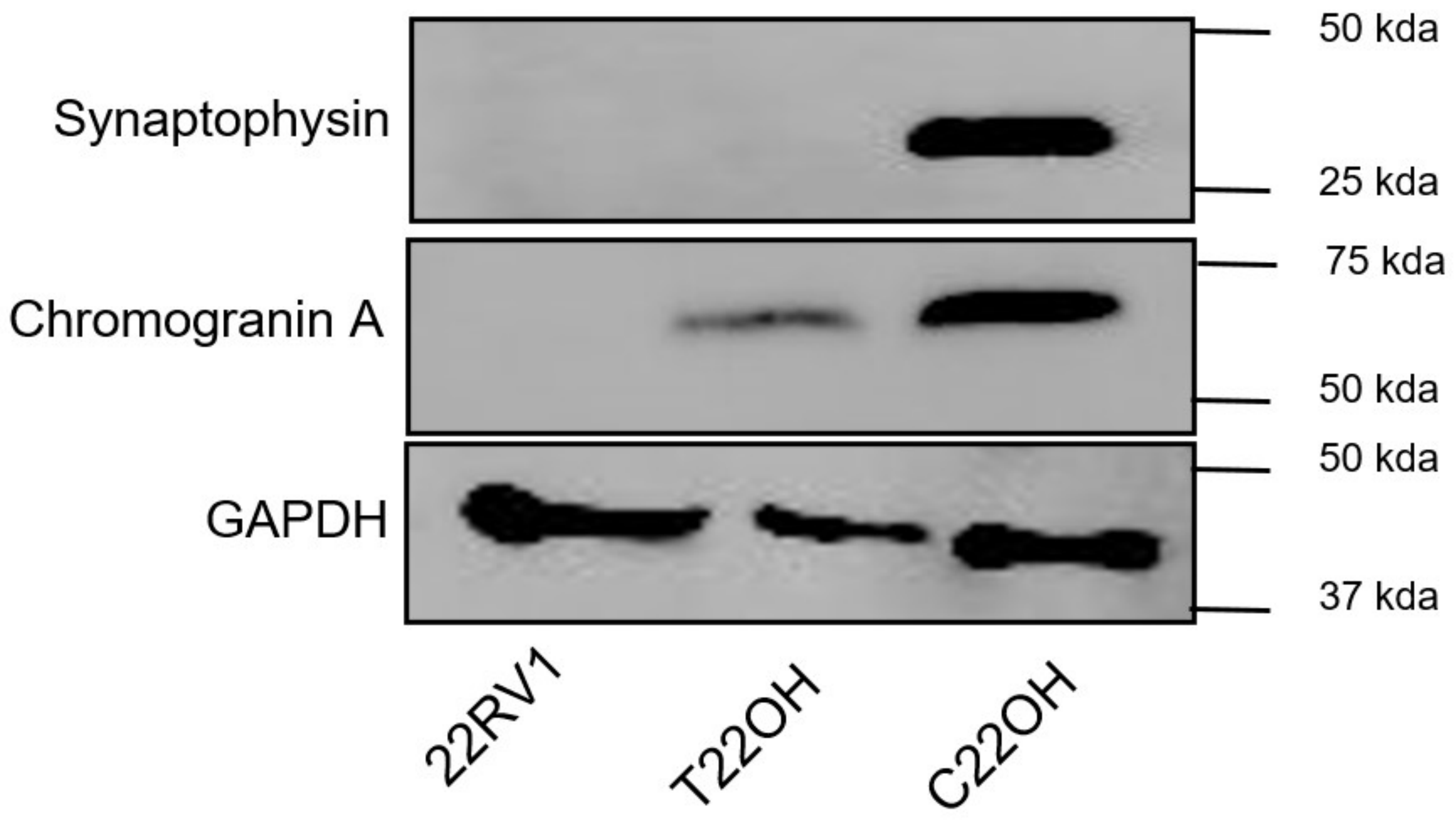

B

	CRPC Vs. Transitional CRPC		Transitional CRPC vs. Classic NEPC	
RSAD2	Fold Change	Adjusted P-Value	Fold Change	Adjusted P-Value
	-1.548	0.0632	**2.15**	**0.000191**

Supplemental Figure 2. C22OH cell line has neuroendocrine features. [A] Western blotting of parental 22RV1, primary tumor cell line T22OH and circulating tumor cell line C22OH looking at two neuroendocrine markers synaptophysin and chromogramin A along with housekeeping gene GAPDH. **[B]** Analysis of GSE199596 assessing classic CRPC (AR positive, neuroendocrine marker negative), transitional CRPC (AR positive, neuroendocrine marker positive), and classic NEPC (AR negative, neuroendocrine positive). Statistics were provided by GEO2.0 analysis and we deemed statistical significance where p-value < 0.05.
