# Supplementary material for: Upregulation of the interferon-inducible antiviral gene RSAD2 in neuroendocrine prostate cancer via PVT1 exon 9 dependent and independent pathways
Source: J Biol Chem. 2025 Feb 28;301(4):108370. doi: 10.1016/j.jbc.2025.108370 (PMC11994405; doi:10.1016/j.jbc.2025.108370)
Supplement: Figure S3 [file mmc3.pdf]

A

	Percent Identity	E-Value
Promoter Region	12 %	0.094
5'UTR	8 %	0.24
CDS	0 %	1.4
3'UTR	15%	0.031
Introns	0%	0.37

B

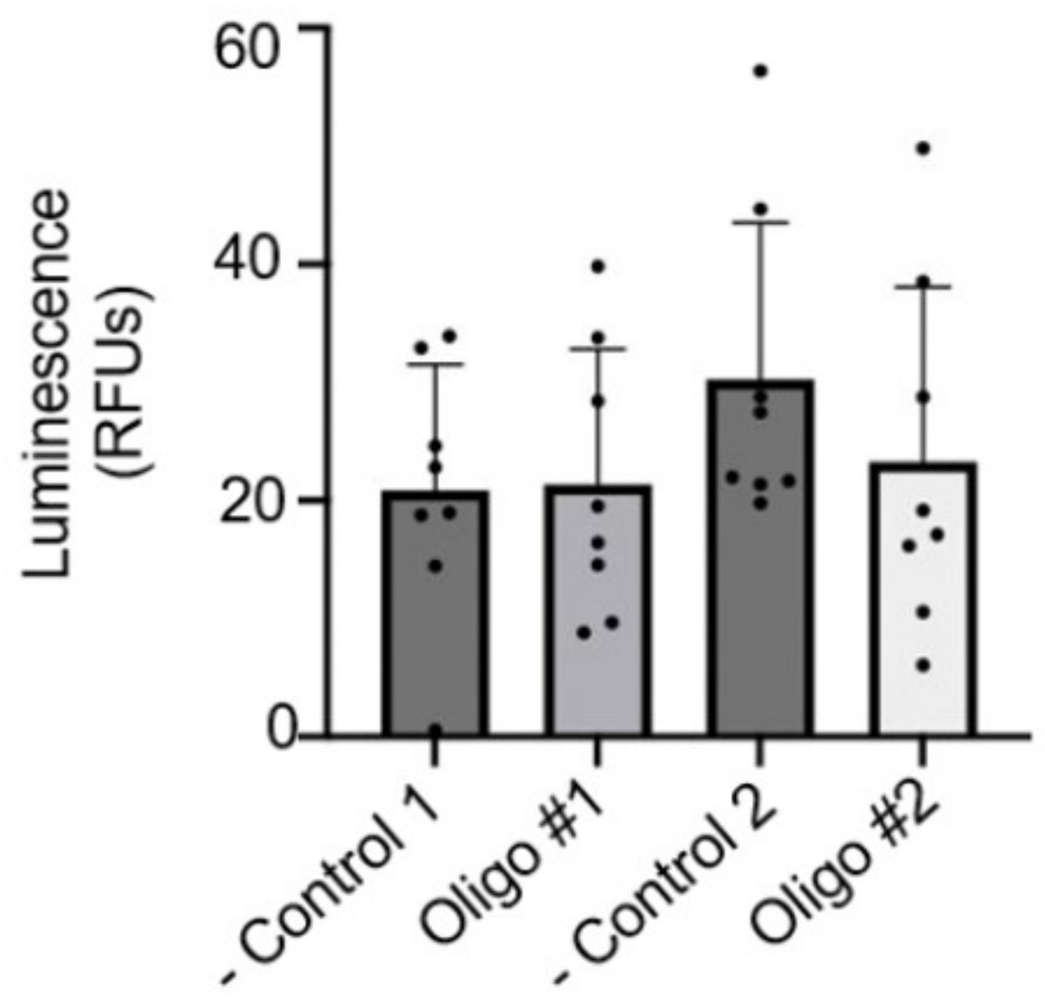

C

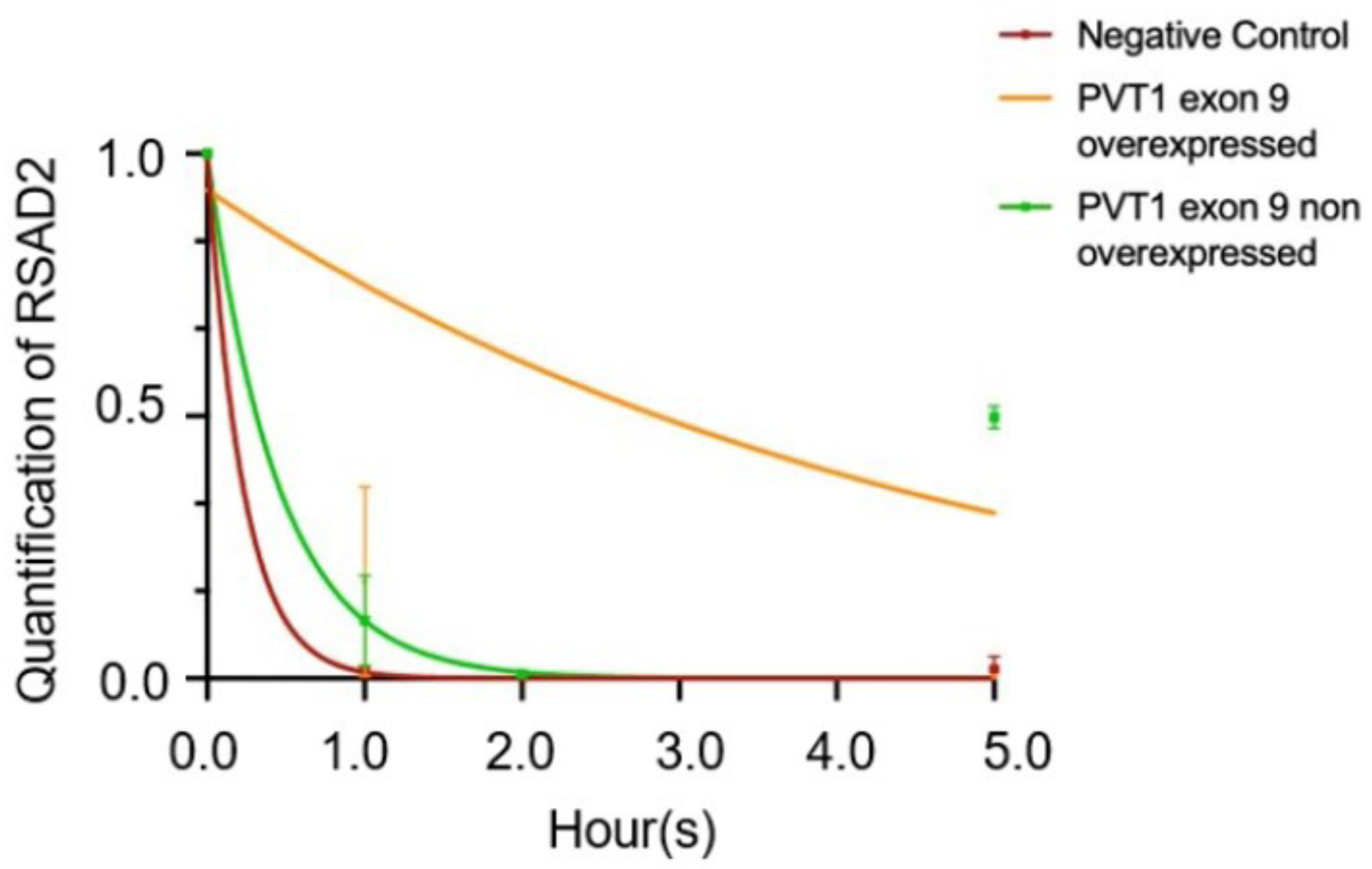

Supplemental Figure 3. PVT1 exon 9 does not directly interact with RSAD2. [A] Sequence alignment of RSAD2 mRNA and PVT1 exon 9 mRNA at various locations on RSAD2 mRNA using BLAST alignment. **[B]** Dual luciferase assay of RSAD2 3'UTR region (three biological replicates) cloned into RWPE1 cell line. PVT1 exon 9 oligo was added with lipofectamine to the cells and luciferase activity was recorded. No significant (p-value < 0.05) binding activity was recorded comparing negative control to PVT1 exon 9 treated cells. **[C]** mRNA decay assay (two biological replicates) showing decay rates of various cell models after being treated at differing timepoints with flavopiridol recorded with RT-qPCR.
