# Supplementary figures and images for: Upregulation of the interferon-inducible antiviral gene RSAD2 in neuroendocrine prostate cancer via PVT1 exon 9 dependent and independent pathways
Source: J Biol Chem. 2025 Feb 28;301(4):108370. doi: 10.1016/j.jbc.2025.108370 (PMC11994405; doi:10.1016/j.jbc.2025.108370)

A

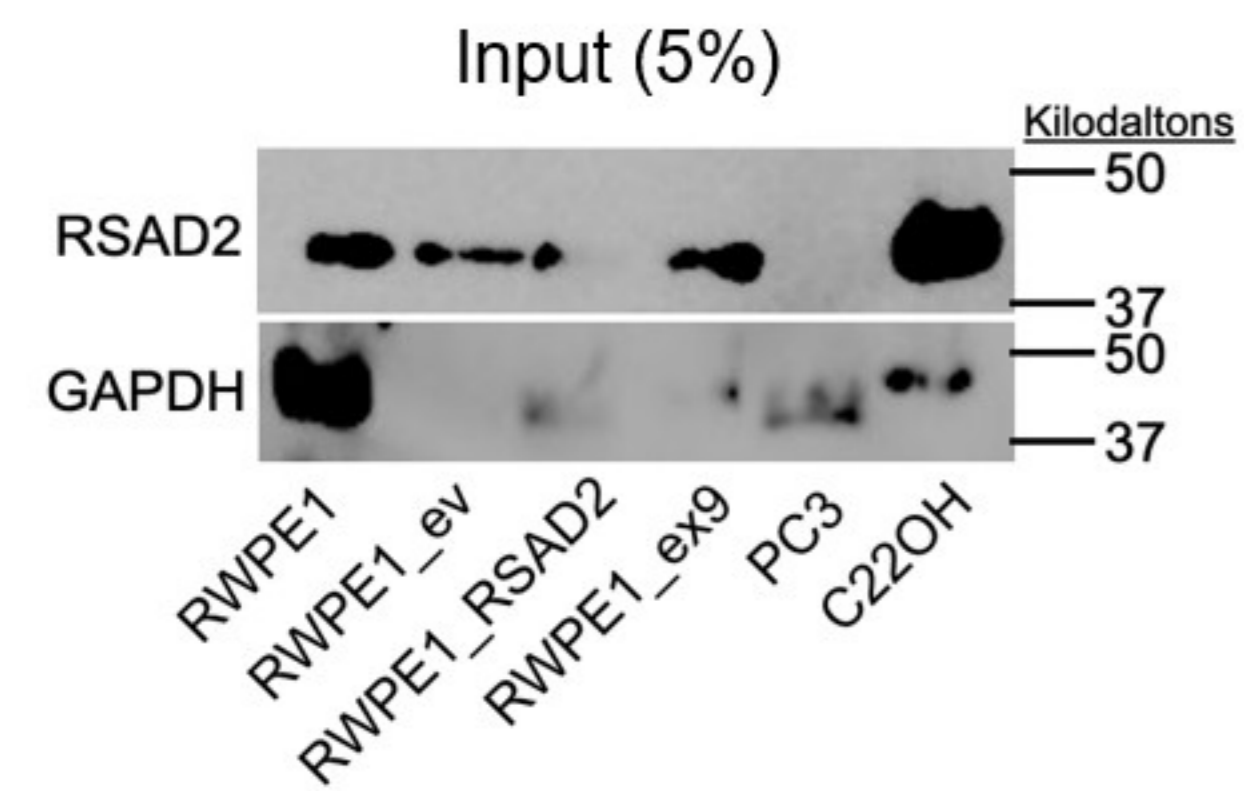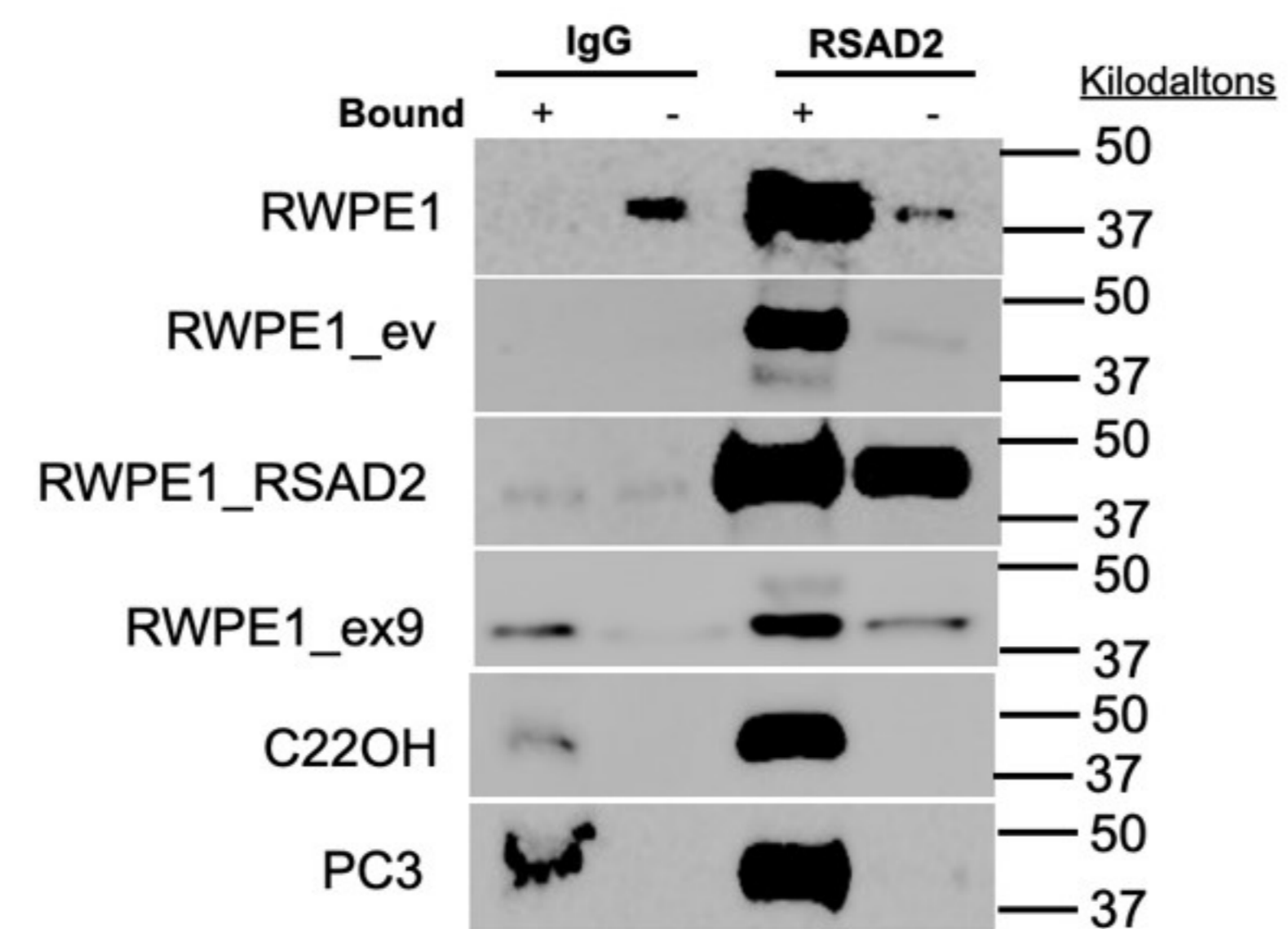

B

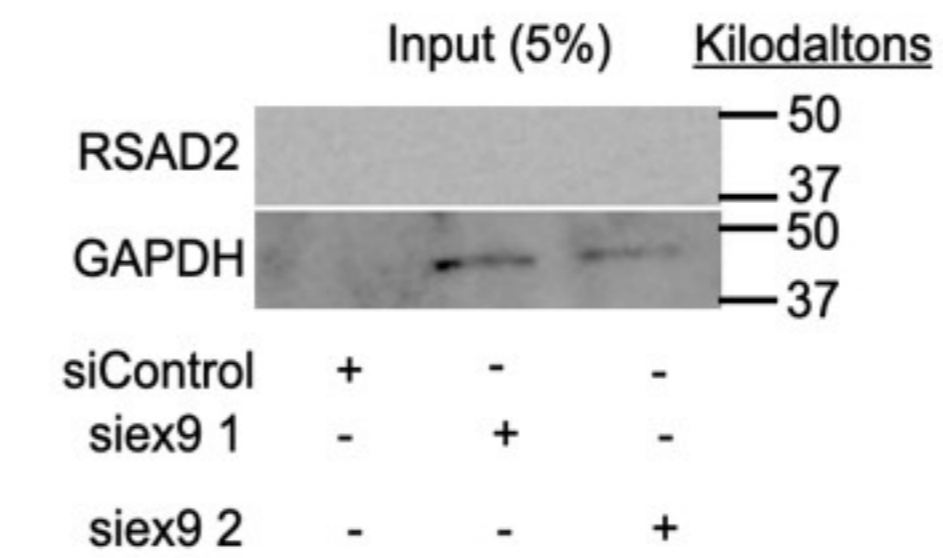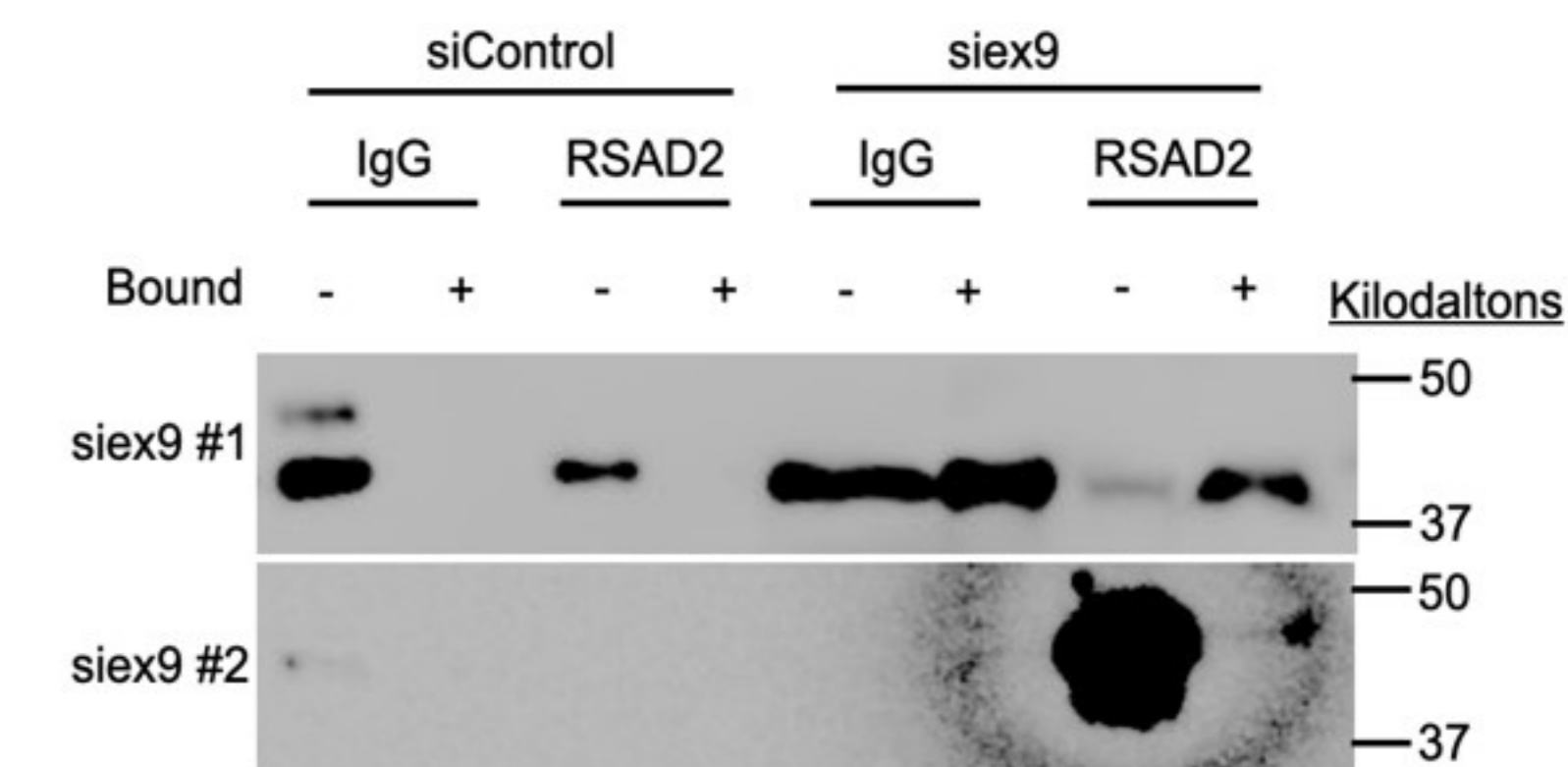

C

### PVT1 Exon 9 Secondary Structure

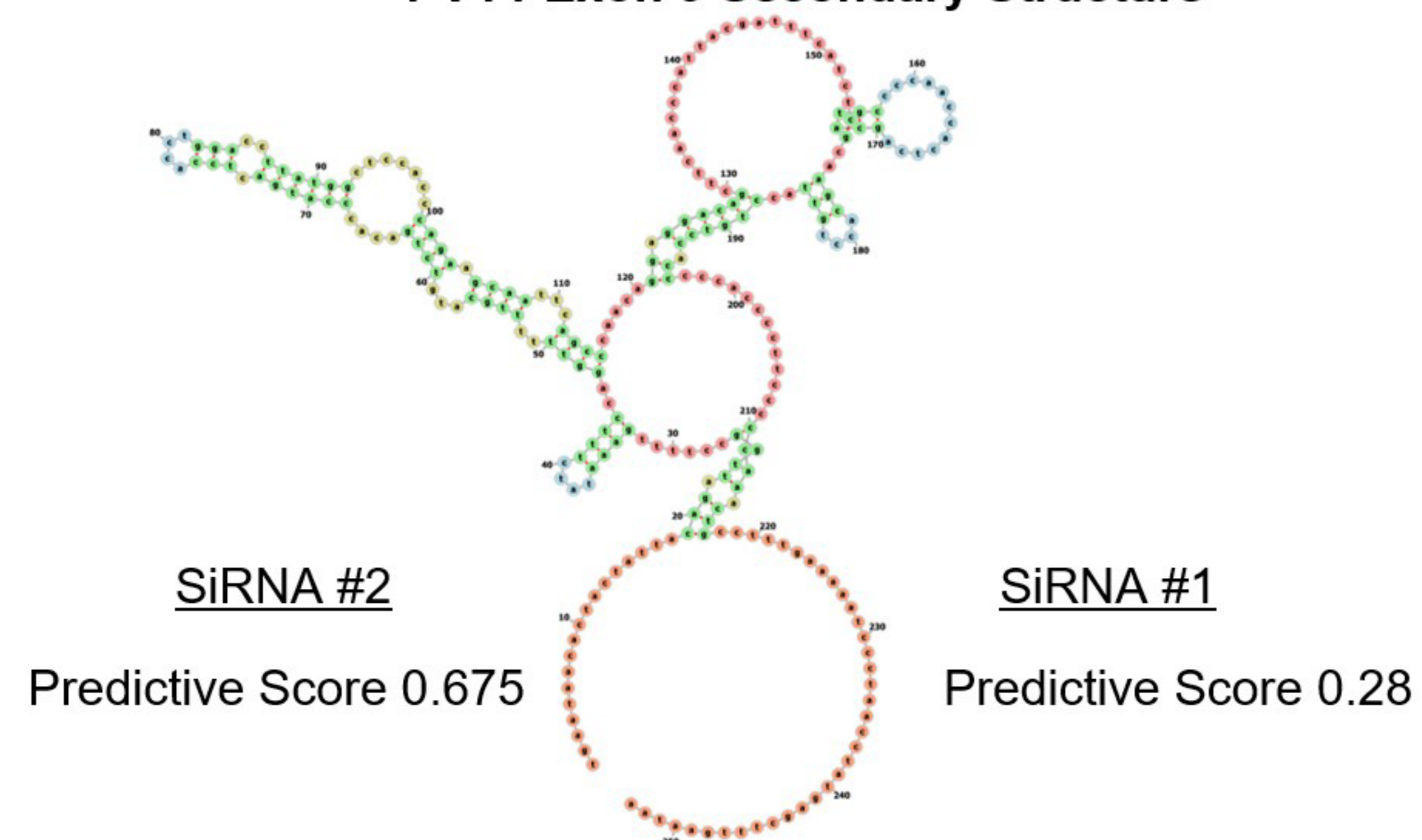

Supplement: Figure S4 [file mmc4.pdf]
