# Supplementary material for: Upregulation of the interferon-inducible antiviral gene RSAD2 in neuroendocrine prostate cancer via PVT1 exon 9 dependent and independent pathways
Source: J Biol Chem. 2025 Feb 28;301(4):108370. doi: 10.1016/j.jbc.2025.108370 (PMC11994405; doi:10.1016/j.jbc.2025.108370)
Supplement: Figure S5 [file mmc5.pdf]

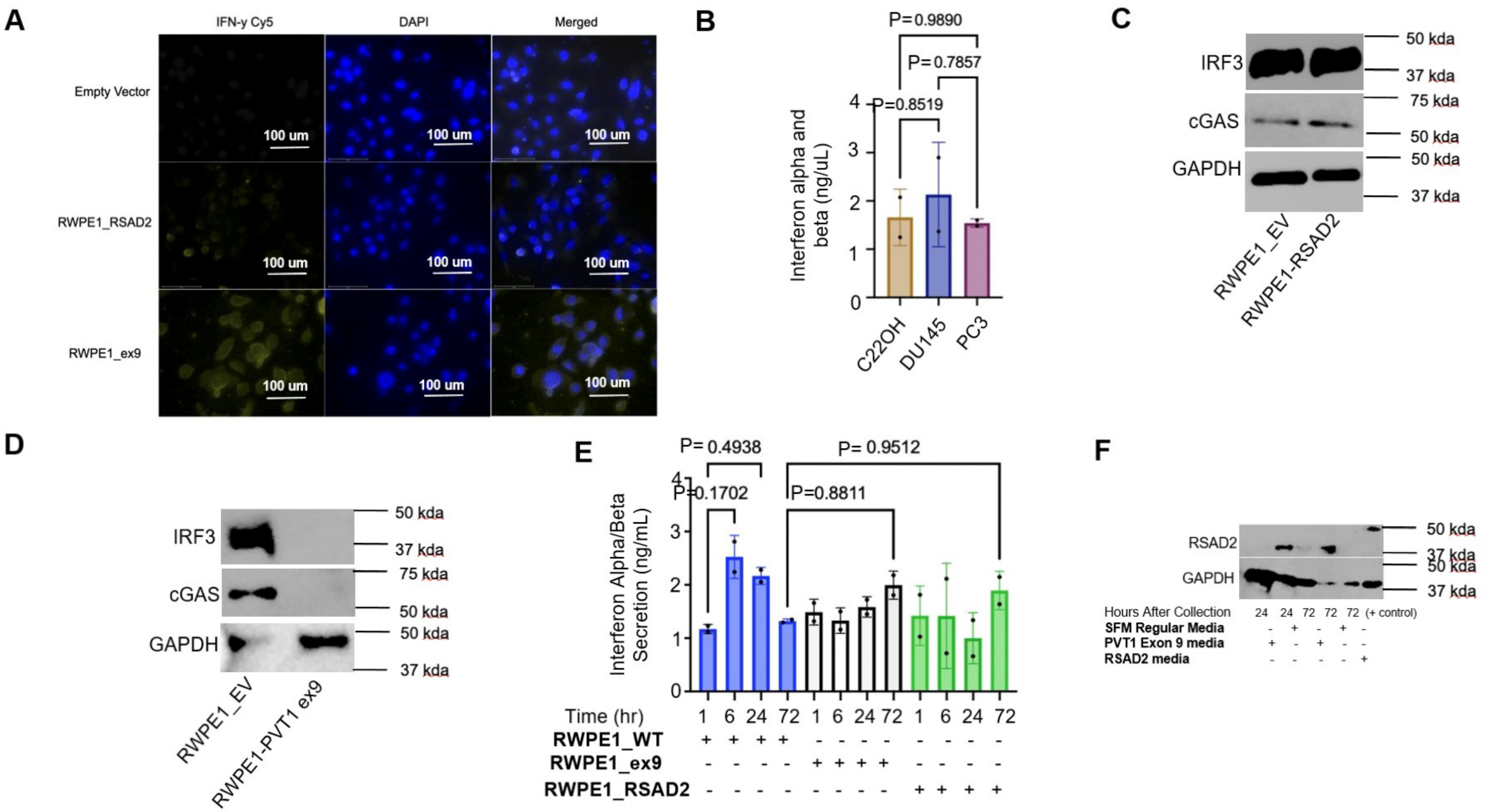

Supplemental Figure 5. Type II interferon signaling is attenuated in PVT1 exon 9 overexpressed NEPC models. [A] Immunofluorescence of interferon gamma in RWPE1_EV, RWPE1_RSAD2 and RWPE1_ex9. Interferon gamma was found in higher intensity intracellularly in the RWPE1_ex9 model compared to RWPE1_RSAD2 and no detection in RWPE1_EV. **[B]** ELISA assay measuring interferon alpha and beta secretion in C22OH, DU145 and PC3 cell lines (n=2). Statsitics were provided by two-tailed students t-test at 95% confidence interval. **[C-D]** Representative western blotting of IRF3 and cGAS in RWPE1_ev, RWPE1_RSAD2 and RWPE1_ex9 using GAPDH as normalized loading control. RWPE1_ex9 cells exhibited loss of IRF3 and cGAS signaling compared to RWPE1_RSAD2 cell line. **[E]** ELISA quantification time course of interferon alpha and beta over 72-hour time period in RWPE1_WT, RWPE1_ex9 and RWPE1_RSAD2 cell lines. Statistics were provided by PRISM software using 2-way ANOVA analysis at alpha value 0.05. **[F]** Representtive western blot of supernatant transfer experiment assessing whether RSAD2 can be induced by PVT1 exon 9 overexpressing cell.
