# Supplementary material for: Upregulation of the interferon-inducible antiviral gene RSAD2 in neuroendocrine prostate cancer via PVT1 exon 9 dependent and independent pathways
Source: J Biol Chem. 2025 Feb 28;301(4):108370. doi: 10.1016/j.jbc.2025.108370 (PMC11994405; doi:10.1016/j.jbc.2025.108370)
Supplement: Figure S6 [file mmc6.pdf]

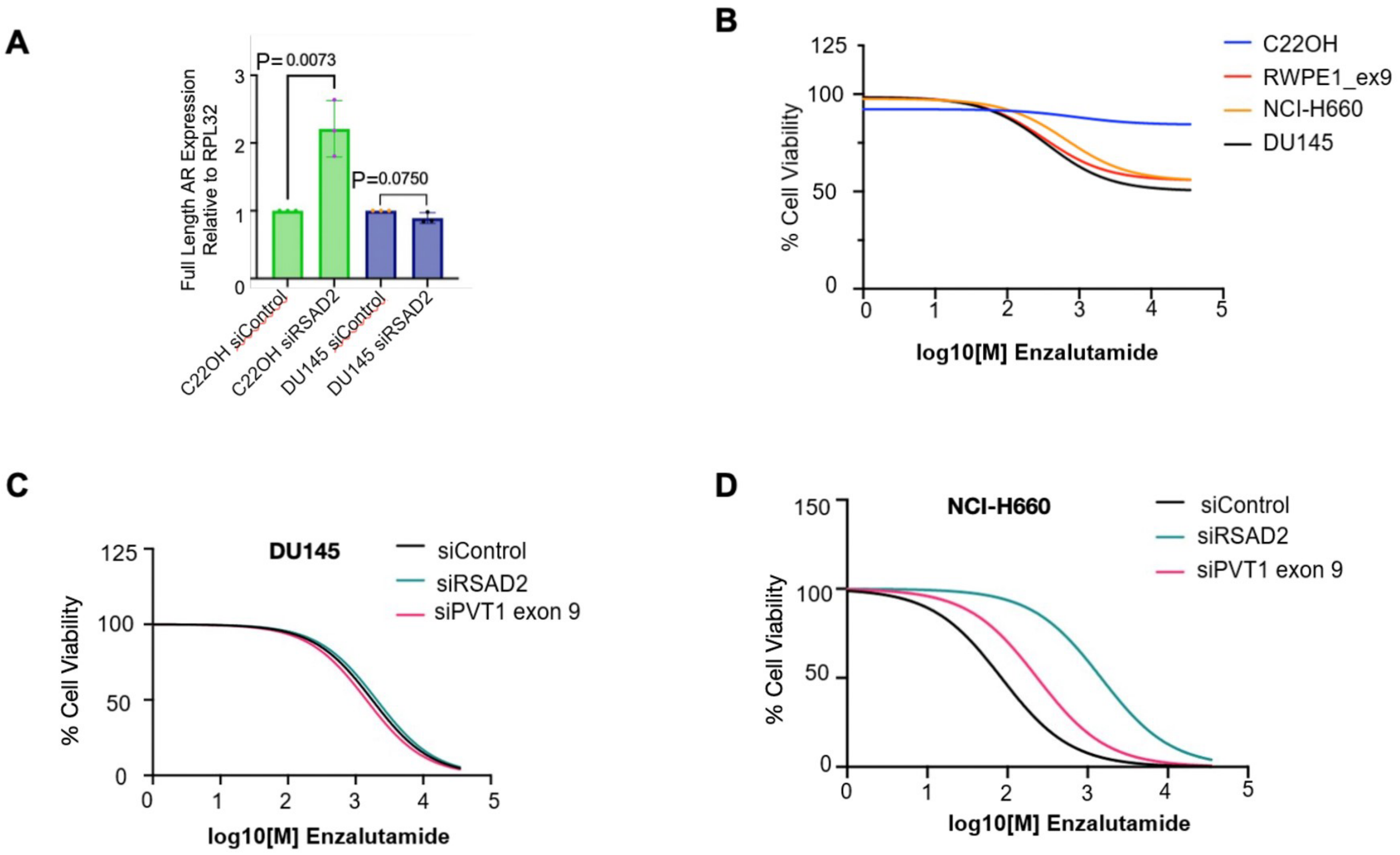

Supplemental Figure 6. Androgen receptor signaling depends on PVT1 exon 9 expression in neuroendocrine prostate cancer models. [A] RT-qPCR analysis of C22OH, DU145 and PC3 cell lines assessing AR expression after RSAD2 knockdown (three biological replicates). Results were normalized to RPL32 housekeeping gene. PRISM provided statistics using two tailed students t-test at 95% confidence interval. **[B]** Baseline cell viability analysis (MTT/WST-1) of C22OH, RWPE1_ex9, DU145 and NCI-H660 assessing sensitivity to enzalutamide (two biological replicates). **[C-D]** Cell viability analysis of NCI-H660 (two biological replicates) and DU145 (three biological replicates) siControl, siPVT1 exon 9 and siRSAD2 assessing sensitivity to enzalutamide. Cells were processed for proliferation compared to normal control using MTT reagent.
