# Supplementary material for: Upregulation of the interferon-inducible antiviral gene RSAD2 in neuroendocrine prostate cancer via PVT1 exon 9 dependent and independent pathways
Source: J Biol Chem. 2025 Feb 28;301(4):108370. doi: 10.1016/j.jbc.2025.108370 (PMC11994405; doi:10.1016/j.jbc.2025.108370)
Supplement: Table S1 [file mmc8.docx]

Supplemental Table 1: Guide Sequences for CRISPRa experiments

| Canonical Guide | Sequence |
| --- | --- |
| gRNA1 PVT1 CP-330 | 5’ACTCCGGCGCGCTTGTAGCG AGG 3’ |
| gRNA 2 PVT1 CP-231 | 5’ TGGGCGACTGCGGCCGCCAA GGG 3’ |
| gRNA 3 PVT1 CP-167 | 5’ GCCTGCTTCCCGGCAGCGCA GGG 3’ |
| gRNA 4 PVT1 CP-99 | 5’GCCGGGACCGAGGACGCACG CGG 3’ |
| gRNA 5 PVT1 CP-32 | 5’ GCGAGCCGCCGTGACGTCAC CGG 3’ |
| gRNA 6 NTC 1 | GATACGTCGGTACCGGACCG |
| gRNA 7 NTC 2 | CTAAGACAGCCAATCACCCA |
| **Alternative Promoter Guide** | **Sequence** |
| gR8 PVT1 exon 9 prom (+) | 5’ TGACCCAGATTTGTAAACAG AGG 3’ |
| gR9 PVT1 exon 9 prom (+) | 5’ GAGAAAGTCGAATGTGCTCG GGG 3’ |
