# Supplementary material for: Upregulation of the interferon-inducible antiviral gene RSAD2 in neuroendocrine prostate cancer via PVT1 exon 9 dependent and independent pathways
Source: J Biol Chem. 2025 Feb 28;301(4):108370. doi: 10.1016/j.jbc.2025.108370 (PMC11994405; doi:10.1016/j.jbc.2025.108370)
Supplement: Table S2 [file mmc9.docx]

Supplemental Table 2: List of siRNAs and primers used.

|  | Forward | Reverse |
| --- | --- | --- |
| RPL32 | CATCTCCTTCTCGGCATCATGG | TGGGAGCCACAAGCTTCTTC |
| RSAD2 | GCGAGATGTGCGCGATAAAC | TCTCGTTCCACTTTCCGCTC |
| PVT1 exon 9 | CATGACTCCACCTGGACCTT | GTGGGCGATGAAGTTCGTA |
| AR | TACCGCCTCACCAAGCTCCT | GCTTCACTGGGTGTGGAAA |
| siPVT1 exon 9 1 | ACCUAUGAGCUUUGAAUAA | UUAUUCAAAGCUCAUAGGU |
| siPVT1 exon 9 2 | UGAAUAACACUACUAUUACAGAUTG | CAAUCUGUAAUAGUAGUGUUAUUCACA |
